# Estimating the Abundance of a Cryptic, Endangered Marsupial, Mala ( *Lagorchestes hirsutus* ) Using Microsatellite and Single Nucleotide Polymorphism Genotyping Panels

**DOI:** 10.1002/ece3.73480

**Published:** 2026-04-29

**Authors:** Deanne Cummins, Kristen Nilsson, Rujiporn Thavornkanlapachai, Kym Ottewell, Cheryl Lohr

**Affiliations:** ^1^ Department of Biodiversity Conservation and Attractions Biodiversity Conservation Science Kensington Western Australia Australia

**Keywords:** arid zone, Australia, faecal DNA, *Macropodidae*, non‐invasive monitoring, secr, spatially explicit capture‐mark‐recapture analysis, species management

## Abstract

Developing high‐quality, non‐invasive population monitoring techniques is important for endangered species that are difficult to trap or susceptible to capture myopathy. One such species is the mala (*Lagorchestes hirsutus,* Central Australian subspecies), an Australian macropod that is extinct in the wild and only conserved inside four predator‐free reserves and on one offshore island. DNA extracted from scat is an effective non‐invasive method to ‘genetically tag’ individuals for capture‐mark‐recapture analyses to estimate population abundance. Microsatellites have previously been successfully used to identify individuals from mala scats, but are prone to genotyping errors, especially when DNA quality is low. Here, we developed an array of 50 highly informative Single Nucleotide Polymorphisms (SNPs) for targeted genotyping on the MassARRAY system to improve efficiency and confidence in individual identification. We compared the accuracy of individual identification using the SNP panel to eleven previously published microsatellite markers and compared estimates of mala abundance derived from each genotyping method. Mala scats were collected in 2020 and 2021 along nine transects within a predator‐free reserve at Matuwa Kurrara Kurrara National Park, Western Australia. The SNP panel had similar amplification success to microsatellites but reduced genotyping error rates. Both genotyping methods showed a similar number of individuals identified and similar patterns in genetic diversity and relatedness across two years. Spatially explicit capture‐recapture modelling using microsatellites produced an estimate of 108 (±37) mala in 2020 and 59 (±22) in 2021. Samples genotyped on the SNP panel produced an estimate of 122 (±45) individuals in 2020 and 81 (±32) in 2021. Both methods indicated a substantial decline in mala abundance from 2020 to 2021, which was likely a lag effect associated with a drought that occurred in 2019.

## Graphical abstract

We developed a species‐specific SNP panel for non‐invasive population monitoring of the mala ( *Lagorchestes hirsutus* , Central Australian subspecies), an Australian macropod that is extinct in the wild, and conserved inside four predator‐free reserves and on one offshore island. We compared the accuracy of individual identification using a species‐specific SNP panel to eleven previously published microsatellite markers, and compared estimates of mala abundance and genetic diversity derived from each genotyping method. The SNP panel had similar amplification success to microsatellites, but substantially reduced error rates, and estimates of abundance were similar for both genotyping methods, both indicating a substantial decline in mala abundance from 2020 to 2021.

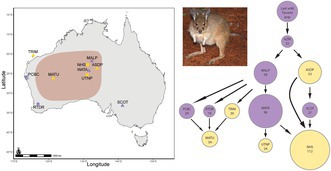

## 1 Introduction

The drastic decline of Australian native mammals since European colonisation has been well documented (Geyle et al. 2018; Wayne et al. 2017), with many species currently relying on intensive conservation management to avoid extinction, such as captive breeding programmes, conservation translocations and reintroductions to offshore islands and to fenced semi‐wild reserves (Moseby et al. 2018). Even as conservation techniques have improved, many of the original factors contributing to population declines remain a serious challenge, particularly the control or eradication of introduced predators, such as red foxes ( *Vulpes vulpes* ) and feral cats ( *Felis catus* ) (Doherty et al. 2022). Similarly, other threats that historically contributed to mammal declines, such as habitat fragmentation and changing fire regimes (Doherty et al. 2022; Morris et al., 2021; Ringma et al. 2018; Wayne et al. 2017), will continue to intensify with climate change (Doherty et al. 2022), increasing the need for intensive conservation management.

Paradoxically, some intensive conservation management actions present another set of unique challenges (Ringma et al. 2018). Fenced reserves, whilst protecting threatened species from excessive predation by introduced predators, prevent the dispersal of growing populations which can result in populations exceeding carrying capacity (Duncan et al., Duncan et al. 2020; Moseby et al. 2018; Treloar et al. 2023), loss of genetic diversity (Ottewell et al. 2014), inbreeding (Ottewell et al. 2014; Rick et al. 2019), and the loss of adaptive predator avoidance behaviours (Harrison et al. 2023). These issues highlight the need for accurate, cost‐effective and timely population monitoring of threatened fauna within fenced reserves to ameliorate these issues. Further, conservation actions need to adapt to changing conditions and this can only be achieved by regularly monitoring a population and evaluating outcomes against conservation objectives.

Non‐invasively collected genetic data is increasingly being utilised in population monitoring, because advances in molecular techniques have made sequencing more efficient and affordable (Kraus et al. 2015). Hence, the techniques are now often logistically and financially more efficient than traditional monitoring techniques via live trapping (Ekblom et al. 2021; Ferreira et al. 2018). Non‐invasive sampling, including camera‐trap data (Thorn et al. 2022), or the collection of DNA from the environment in the form of scat, urine, feathers, or hair for genetic tagging (Thompson 2004), are particularly useful for cryptic species that are difficult to trap, or suffer from capture myopathy: a potentially lethal trap‐related stress (Cowen et al. 2022). For species that lack unique external features, faecal DNA (or scat DNA) analysis offers the potential to estimate population abundance by identifying individuals from their genetic fingerprint, the unique set of genotypes at multiple loci, obtained by analysis of epithelial cells retained on the surface of the scat (Cowen et al. 2022). However, DNA recovered from scats is often of low quantity and quality (Cowen et al. 2022; Dziminski et al. 2021; Kraus et al. 2015), sometimes making individual identification challenging.

Microsatellite markers have successfully been used for individual identification (Ottewell et al. 2020) and population abundance estimation of a few threatened native Australian mammals from scat DNA (Cowen et al. 2022; Dziminski et al. 2021; Treloar et al. 2023), and to estimate the abundance of other mammals around the world (Ferreira et al. 2018; Fuller et al. 2016; Goode et al. 2014; Morin et al. 2016, 2018). The advantage of microsatellite markers includes their multi‐allelic nature, meaning that the statistical power to identify individuals can be achieved with relatively few markers (Fabbri et al. 2012). The cost of developing markers is relatively low, and markers can often be selected from closely related species (Fabbri et al. 2012). However, microsatellite markers are prone to laboratory artefacts such as stutter peaks, false alleles and allelic dropout making them difficult to score reliably, especially when DNA is low in quality or quantity (Hoffman and Amos 2005). While these issues can be mitigated through a ‘multi‐tube’ approach (where DNA samples are amplified several times independently, creating a consensus genotype), by manually assessing genotypes to ensure correct and consistent calls (Frantz et al. 2003; Taberlet et al. 1996), or by strategically repeating a portion of samples to estimate genotyping error rates (e.g., Ottewell et al. 2020), these approaches can be time‐consuming and costly. Moreover, comparing results between years, technicians and/or laboratories in long‐term systematic monitoring programmes requires further standardisation to ensure accuracy (e.g., Ellis et al. 2011). As such, the development of Single Nucleotide Polymorphism panels (SNP panels), genotyped via automated platforms (e.g., MassARRAY, Ampliflour and Fluidigm (see Carroll et al. 2018)), for use in population monitoring is becoming a popular alternative.

SNP panels combine a small number of informative SNP loci, multiplexed to function in a single genotyping array (Kraus et al. 2015). SNP markers allow genotyping of a greater number of loci for delineating individuals in a single assay, and their biallelic nature makes it easy to automate genotyping. SNP markers rely on the presence/absence of pre‐determined alleles, which reduces laboratory handling, lowers error rates (Thavornkanlapachai et al. 2024), and enhances reproducibility across projects (Ekblom et al. 2021; Kraus et al. 2015; von Thaden et al. 2017). For example, a panel of approximately 100 moderately polymorphic SNPs (minimum allele frequency of 0.2) provides sufficient statistical power to estimate pedigree relationships equivalent to 16–20 microsatellites, and fewer SNPs are required if they are highly polymorphic (Glaubitz et al. 2003). Once a SNP panel has been designed, successive genotyping of many samples is relatively cheap and efficient, making them cost‐effective for ongoing or large‐scale population monitoring projects (von Thaden et al. 2017). Although many studies discuss the benefits of using a panel of SNPs over microsatellites for accurate individual identification (Ekblom et al. 2021; von Thaden et al. 2017), few studies directly compare the two methods using a common set of samples (Scholz et al. 2024).

**FIGURE 1:**
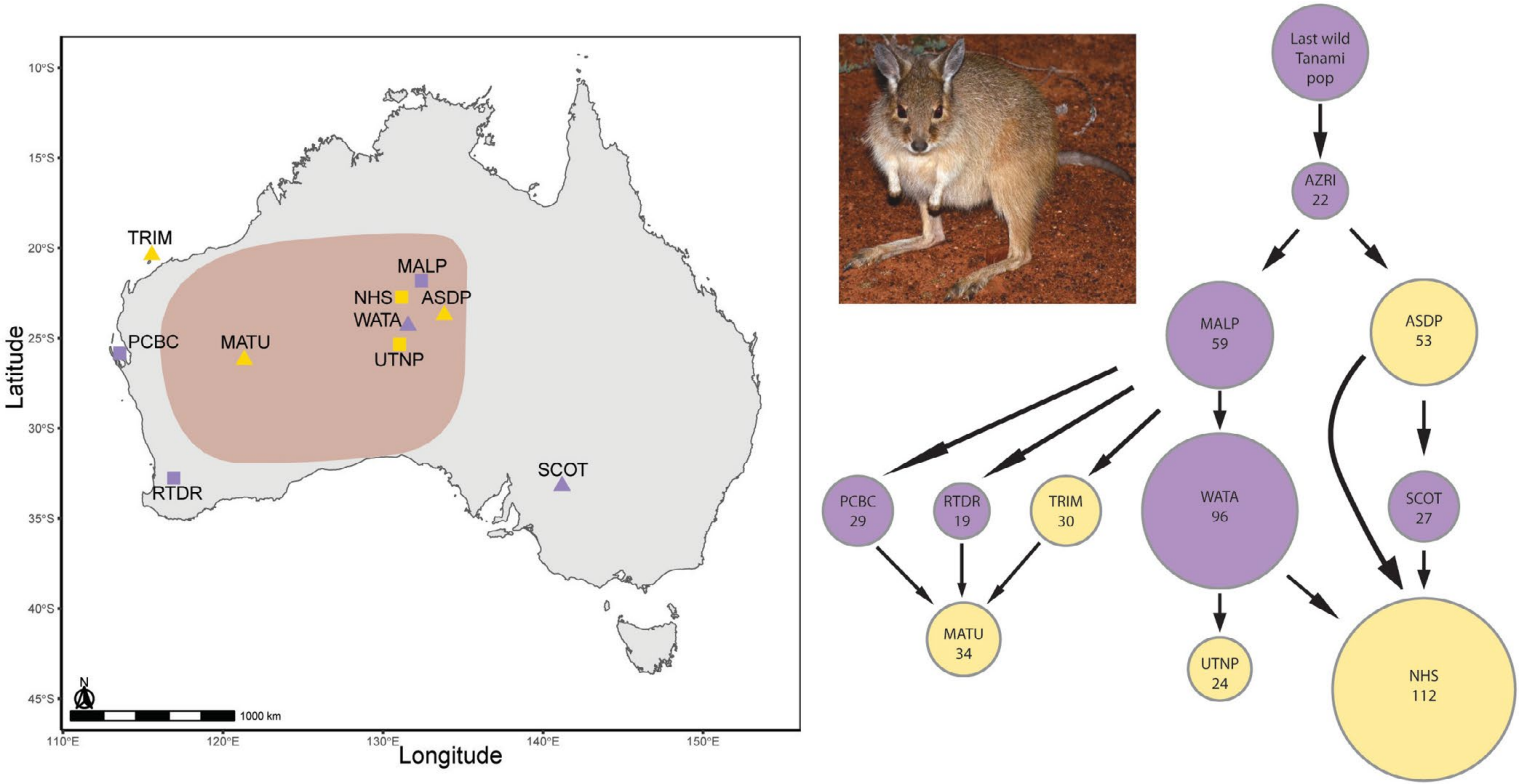
Extant and extinct mala populations across Australia, overlaid on top of the historical distribution of the species, along with a flow diagram of mala translocation movements. Extant populations are shown in yellow and extinct populations are shown in purple on both the map and the flow diagram. Triangles represent populations used in the design of the MassARRAY panel and squares represent populations that were not included. Location codes are: Trimouille Island (TRIM), Peron Captive Breeding Centre (PCBC), Return to Dryandra captive breeding program (RTDR), Matuwa Kurrara Kurrara National Park (MATU), Mala Paddock Lander River (MALP), Newhaven Sanctuary (NHS), Watarrka National Park (WATA), Uluru National Park (UTNP), Alice Springs Desert Park (ASDP), Australian Wildlife Conservancy's Scotia Reserve (SCOT), and Arid Zone Research Institute (AZRI). The number of founding individuals of each population is represented underneath each population code in the flow diagram, and the size of each circle provides a visual representation of the number of founders.

Mala is an undescribed mainland subspecies of the rufous hare wallaby, a small endemic macropod ( *Lagorchestes hirsutus* spp.) listed as Vulnerable by the IUCN (Burbidge and Woinarski 2016). The mala is one of two extant subspecies of rufous hare wallaby. The other (*L. hirsutus bernieri*) includes both island populations (Bernier Island, Western Australia and Dorre Island, Western Australia) (Eldridge et al. 2019). Mala were once abundant across the central Australian arid zone (Pearson, 1989), but are now extinct in the wild on the mainland, largely due to predation by introduced species (the European red fox and feral cats), changes in fire regimes (Richards 2012), and habitat destruction and alteration (Saxon 1983). The species has been conserved through introduction to one offshore island, Trimouille Island in the Montebello Archipelago (Van Dyck 2008), and to a series of predator‐free fenced reserves and captive breeding programmes on the mainland (Richards 2012; Treloar et al. 2023), only four of which remain operational (Figure 1). Multiple conservation introduction and reintroduction attempts of mala (Table S1) have created a series of population bottlenecks for the species (Bouzat 2010). The last wild mala population (est. < 200 individuals) was located in the Tanami Desert, in the Northern Territory (Bolton and Latz 1978; Burbidge et al. 1988; Parker 1973), from which the first captive population at the Arid Zone Research Institute was established in 1981 with 22 founders (Lundie‐Jenkins 1993b).

Matuwa Kurrara Kurrara National Park (hereafter referred to as Matuwa) is one of four introduced predator‐free reserves that protect mala (Treloar et al. 2023). The reserve covers 1100 ha of habitat in the arid region of central Western Australia and protects two other threatened Australian marsupials, burrowing bettongs (mitika, boodie, *Bettongia lesueur* ) and golden bandicoots ( *Isoodon auratus* ) (Lohr et al. 2021), along with a reintroduced population of brushtail possums ( *Trichosurus vulpecula* ) (Semple et al. 2020), and recovering populations of mulgara (*Dasycercus blythi*) (Read et al. 2023) and other small native mammals (Lohr 2019). The Matuwa mala population was established using 34 individuals from Trimouille Island in 2011, 12 animals from the Return to Dryandra captive breeding program in 2012, and 22 animals from Peron Captive Breeding Centre in 2013 (Lohr 2019; Sims et al. 2017).

Previous methods of population monitoring of the Matuwa mala included targeted trapping and spotlight monitoring, which suggested that the population was small but stable given individuals' breeding activity and body condition (Lohr 2019; Sims et al. 2017). However, these methods did not provide sufficient data for accurate abundance estimates, which is crucial for assessing ongoing translocation success (Lohr 2019). Mala are highly susceptible to capture myopathy (Morris et al. 2004), a condition where potentially fatal physiological imbalances develop due to extreme stress, making intensive capture‐recapture programmes unsuitable for regular population monitoring. More recently, mala abundance was estimated from scat samples collected across two seasons in 2019, using microsatellites, showing approximately a two‐fold increase in abundance in the Matuwa reserve since establishment (Treloar et al. 2023). Further attempts to estimate mala abundance using scat DNA and microsatellites suggested the mala population declined sharply in 2021. This contrasting result raised two questions about the accuracy of microsatellites for estimating mala abundance from scat DNA: (1) whether the accuracy of individual identification was negatively impacted by a high rate of genotyping errors; or (2) if the microsatellite markers were variable enough to identify individuals of a subspecies that has likely suffered substantial losses in genetic diversity over time, due to an initial bottleneck, followed by subsequent serial founder events as new translocated populations were established (fenced or captive), both of which can erode genetic diversity over time (White et al. 2018).

In this study, we described the development of 50 novel SNP markers designed for the MassARRAY genotyping platform for individual identification of mala from scat DNA. Our primary aim was to compare the effectiveness of individual identification using microsatellites versus the SNP panel and assess the impact of these techniques on downstream estimates of abundance from mark‐recapture analysis. Our secondary aim was to quantify changes in mala abundance over time and investigate whether abundance varied across habitat type in the Matuwa reserve, and determine whether the observed trend was consistent between the two genotyping techniques. We expected the SNP panel to have a higher amplification success than microsatellites, lower error rates, and a greater accuracy of individual identification, which would subsequently improve abundance estimates.

## 2 Methods

### 2.1 Sample Collection and DNA Extraction

Mala scat samples were collected from Matuwa over three days in September 2020 (*n* = 109), and one day in September 2021 (*n* = 95). The sampling area consisted of 99 sampling points which were spaced at 150 m intervals along nine parallel transects, approximately 500 m apart. Scats were collected within a 5 m radius of each sampling point. Multiple faecal pellets were collected into one vial if they occurred in a tight pile and were likely to have been deposited by a single animal (single capture event); if not, then they were collected in different vials to avoid cross‐contamination. We aimed to collect fresh scats, characterised by a smooth and shiny surface, to maximise the probability of DNA availability and amplification (Triggs, 2004). Collection forceps were sterilised between each sample collection, using household bleach and distilled water to avoid contamination, and scat samples were stored in sterile 5 mL plastic vials at −18°C until DNA extraction (Piggott et al. 2006). DNA was extracted from scat samples using the Mag‐Bind Stool DNA 96 Kit following the manufacturer's standard protocol (Omega Bio‐Tek, Norcross, GA, USA). To maximise the amount of species‐specific DNA and limit the amount of digestive material obtained from each scat sample, the outer layer of the pellet was gently scraped using a scalpel and used as starting material in DNA extractions to target mala epithelial cells. The whole pellet was used if it was too small. We aimed to genotype the same scats using both SNP and microsatellite methods; however, not all 204 scat DNA samples were available at a high enough quality or volume to be included in both methods, and in some cases samples failed genotyping via one method but not the other. We genotyped 141 samples using microsatellites, 152 samples using SNPs and 121 samples using both methods (see details below). Microsatellite genotyping was performed first and for samples where the volume of DNA leftover was insufficient, DNA was extracted from stored scat samples (additional pellets collected from the same individual), which by that stage, were 3–4 years old (*n* = 96).

### 2.2 Identification of SNP Loci, MassARRAY SNP Panel Design and Validation

The mala MassARRAY was designed using a single nucleotide polymorphism (SNP) dataset, originally generated for population genomic assessment of the species (Ottewell, unpubl. data), via ddRAD sequencing of mala tissue samples collected from five mala populations, including Matuwa, Western Australia (*n* = 16, collected 2012–2017), Trimouille Island, Western Australia (*n* = 13, 2011–2016), Watarrka National Park, Northern Territory (*n* = 10, 2017), Scotia Wildlife Sanctuary, New South Wales (*n* = 23, 2017) and Alice Springs Desert Park, Northern Territory (*n* = 11, 2017). DNA from each tissue sample was sent to the Australian Genome Research Facility (AGRF) for library preparation and sequencing. Briefly, DNA was digested with two restriction enzymes (PstI and NlaIII), ligated with unique barcoded adapters, purified, and size selection (280–375 bp) of the reads was performed on the Blue Pippin (Sage Science). Indexed primers were used to PCR amplify libraries, which were then sequenced on the Illumina NextSeq500. Single end reads were demultiplexed, cleaned and filtered based on quality scores (< 30) following the *process_radtags* pipeline in Stacks v2.68 (Catchen et al. 2013), and truncated to a final length of 142 bp, since sequencing quality declined after this point (FASTQC; Andrews 2010). Demultiplexed reads were aligned to the tammar wallaby ( *Macropus eugenii* ) reference genome (v7) using BWA‐mem2 v2.2.1 (Vasimuddin et al. 2019), with high mapping success (~99%). To identify and call SNPs, unique alignments were passed to the *ref_map* pipeline of Stacks.

The SNP panel was designed with Matuwa as the focal population; however, samples from additional mala populations were included to provide the capability of using the panel to monitor mala collectively across sites. Highly informative candidate SNP loci for individual identification were selected following quality control filtering of SNP data using a custom R script (R v4.3.1; R Core Team 2020; sourced from Thavornkanlapachai et al. (2024)). Briefly, loci with more than one SNP per sequence, which had SNPs located within the 30 base pairs of flanking sequence, and loci with low read quality (Q score < 30) and shorter than 50 base pairs were removed (Table S2). Additionally, using functions from ‘*dartR*’ v2.9.7 (Gruber et al. 2018), loci that had relatively low (< 8) or high (> 100) average sequence read depth, low call rate (loci < 0.78, individuals < 0.8), and highly similar, potentially paralogous, sequences (proportion < 0.2 base pair differences) were removed. Using the gl.filter.maf function, a higher minor allele frequency (MAF) threshold was applied to the focal population (Matuwa) to target loci that were highly variable and informative in this population. Loci that had a MAF of less than 0.34 in the Matuwa population and less than 0.14 in all other populations (Alice Springs Desert Park, Scotia Sanctuary, Trimouille Island, and Watarrka National Park) were removed. Additionally, loci that deviated significantly from Hardy–Weinberg equilibrium in each population (HWE; < 0.05) and loci with extremely low or high heterozygosity across all populations (He; 0.4 < x > 0.6) were also removed (Table S2). Pairs of loci that were likely in linkage disequilibrium (*r* ^*2*^ > 0.3) were removed using ‘*SNPRelate*’ v1.38.0 (Zheng et al. 2012).

We estimated the probability of identity (P_ID_) for unrelated and related individuals in the 86 resultant loci using GENALEX (version 6.51b2, (Peakall 2012); Fig. S1). Sequences of all candidate loci were sent to AGRF for multiplexing analyses and primer design using the Assay Design Suite (v2.2, Agena Bioscience, San Diego, CA, USA). Fifty of 86 candidate loci were optimised for the MassARRAY SNP panel (hereafter referred to as the SNP panel), the largest panel size possible for this system.

To validate the genotyping success and accuracy of the SNP panel, 24 tissue (10 ng/μL) and 12 scat (unknown concentration; collected 2021) DNA samples were sent to AGRF for genotyping. Where possible, tissue DNA samples were selected from the samples used to generate the ddRAD data, covering Scotia Sanctuary (*n* = 3) and Trimouille Island (*n* = 2). Where there was not enough DNA left in samples used for ddRAD, extra samples from each of the following populations were included: Matuwa (*n* = 6), Trimouille Island (*n* = 6), Peron Captive Breeding Program (*n* = 3), Scotia Sanctuary (*n* = 1), Watarrka National Park (*n* = 1) and Return to Dryandra captive breeding program (*n* = 2). Five replicates (3 tissue, 2 scat) were randomly selected to assess genotyping error rates, whereby the same DNA extract was genotyped twice.

Genotyping of loci on the SNP panel was carried out via the MassARRAY system (Agena Biosciences, San Diego, CA, USA) at AGRF. The MassARRAY system allows efficient, targeted genotyping of highly multiplexed reactions under standard PCR conditions, meaning up to 50 DNA variants can be genotyped side‐by‐side in a single run (Ellis and Ong 2017). Amplification and extension reactions were performed with 1 μL of extracted DNA, using the iPLEX Gold Reagent Kit (Agena Bioscience, San Diego, CA, USA), following manufacturer instructions. SNP genotypes were determined by mass spectrometry and called using the MassARRAY TyperAnalyzer 4.1 software (Agena Bioscience, San Diego, CA, USA). Tissue samples that were sequenced using ddRAD and MassARRAY sequencing (*n* = 5) were used to check if genotypes obtained from the SNP panel were consistent with those obtained via ddRAD. To this end, genotypes for each sample produced by the SNP panel were cross‐checked with the genotypes obtained from ddRAD sequencing for the loci included in the panel.

### 2.3 Genotyping and Identification of Individuals From Matuwa Mala Scats

#### 2.3.1 SNP Panel

Following validation, we genotyped 152 scat DNA samples (*n* = 89, 2020 and *n* = 63, 2021) with the newly designed mala SNP panel. We calculated average amplification rates across samples and loci, removed samples and loci with very low amplification success (< 0.2), and estimated allelic dropout (Pompanon et al. 2005) across 15 (10%) technical replicates. Technical replicates were randomly chosen DNA extracts that were genotyped twice. Consensus sequences were manually created from each pair of technical replicates. Genotyping information was “filled in” from one replicate if missing from the other, and when genotypes differed between the replicates, information from the replicate with higher amplification success was kept. The SNP results were then processed in a custom R package ‘*ScatMatch’* (Huntley 2021), which facilitates sample and locus filtering before clustering samples based on the number of allelic mismatches between them. A series of visualisations and clustering analyses allow the user to choose a threshold (*h*) of the maximum number of allelic mismatches between samples for them to be grouped and considered as the same individual (Huntley 2021). Mismatches did not include missing data.

We identified the number of ‘unique’ individuals from the remaining samples and loci using multiple processes. First, the data were cleaned by removing samples and loci with low amplification rates (< 0.6), since samples with low amplification rates are likely to be older and contain more errors (Schultz et al. 2018). We looked for an association between amplification success and locus error rate (both metrics were calculated using *ScatMatch*), using a Pearson's test for correlation (*‘cor.test’*) in the R package *‘stats’*. We trialled a few combinations of sample and locus amplification thresholds (ranging from 0.6 to 0.9) to retain only high‐quality samples in the analysis. We employed hierarchical cluster analysis in *ScatMatch* with the function ‘*hclust*’, whereby the algorithm initially assigns each sample to an independent cluster and then proceeds iteratively to join the most similar clusters until all samples are assigned to a single cluster. To determine the maximum number of allele mismatches or ‘*h*’ for which samples should be grouped as the same individual, we used *ScatMatch* functions such as ‘*elbow_plot*’, ‘*heat_plot*’ and ‘*missasign*’ to visualise clustering outcomes (Huntley 2021).

#### 2.3.2 Microsatellites

We genotyped 141 scat DNA samples with 11 previously published microsatellite markers developed for mala (Treloar et al. 2023). Briefly, polymerase chain reactions (PCR) were carried out twice for each sample using the Qiagen Multiplex PCR Plus kit (Qiagen, Hilden, Germany), and the same multiplex combinations as previously used (Treloar et al. 2023). Each multiplex reaction contained 4 μL Qiagen mastermix, 1 μL primer mastermix (2 μM of each primer), and 4 μL DNA and was run on an Eppendorf Mastercycler (Eppendorf, Hamburg, Germany) using the cycling conditions described in Treloar et al. (2023). Amplification products from the two independent PCR runs were separated on an ABI 3730 capillary sequencer using a commercial service (Western Australia State Agricultural Biotechnology Centre). Fragment sizes were determined using an internal size standard (LIZ500, Applied Biosystems, Waltham, MA, USA) and scored in GeneMapper v.6.0 software (Applied Biosystems).

We manually calculated average amplification rates across all loci and samples (including replicates) and removed those with low amplification success (< 0.2). We then subset samples to those genotyped using both microsatellites and SNPs (*n* = 121) and estimated genotyping error rates (allelic dropout and false alleles; Pompanon et al. 2005) across replicates. Error rates were calculated for each locus and then averaged across loci. To ensure a more equitable assessment of amplification success relative to the SNP panel, we calculated the average amplification for each replicate group (R1 and R2) independently. We also looked for an association between amplification success and locus error rate, using a Pearson's test for correlation in the R package *‘stats’*.

Consensus genotypes for each scat sample were called from the comparison of the two replicates. Where replicate genotypes differed, the genotype of the sample with a higher amplification rate was chosen as the representative genotype. If genotype information was missing for one replicate, it was “filled in” by the other replicate, provided that the sample amplification was > 0.2. We then followed the same processing steps as the SNP dataset.

Microsatellite genotypes cannot be imported directly into *ScatMatch* as the package has been designed for bi‐allelic markers. Thus, the filtering of microsatellite data, based on the same sample and locus amplification thresholds (0.6–0.9) as were applied to the SNP data, was done manually in R. The filtered data could then be used in the *ScatMatch* function ‘*hclust*’ for a hierarchical clustering analysis. As above, an elbow graph, heat map, and misassignment graph were generated at each combination of filtering thresholds to choose *h*.

#### 2.3.3 Comparing MassARRAY and Microsatellites

We compared the assignment of scat samples to unique individuals based on filtered genotypes obtained from the SNP panel (‘SNP dataset’) versus microsatellite scoring (‘MSAT dataset’; Table S3). Individual assignment was considered a match if scat samples were clustered similarly (i.e., the same set of samples were assigned to a group or a single scat assigned as a unique group (‘singleton’)) by both methods. Conversely, a mismatch was identified where samples were clustered by one method but assigned to different group(s) or as singletons, by the other method. We investigated the effect of amplification success and locus error rate on the assignment of scat samples to matching groups, using a binomial generalised linear mixed model. We used the ‘*glm*’ function from the R package ‘*stats*’ to construct the model, with ‘matching samples’ as the response variable and amplification success, locus error rate, and microsatellite false allele rate as the main effects. Samples or sample groups that matched were coded as a binary variable, where ‘1’ represented sample assignment considered to match between genotyping methods, and ‘0’ denoted mismatched samples. Measurements of amplification success and locus error rates were calculated for both genotyping methods and were tested independently as main effects, apart from the false allele rate which could only be calculated for microsatellites.

### 2.4 Population Genetic Analysis

To undertake population genetic analyses, we selected a sample with the highest amplification rate as a representative genotype for each ‘individual’. Where multiple scat samples collected in different years were assigned to the same individual, the sample collected in 2021 was included in an attempt to balance sample size across the two years. For clarity, individuals identified using the SNP panel will hereafter be referred to as “SNP Individuals”, and individuals identified using the microsatellites will be referred to as “MSAT Individuals”.

Genetic diversity estimates, such as expected and observed heterozygosity, average number of alleles and the inbreeding coefficient were calculated using GENALEX version 6.51b2, (Peakall 2012), for the SNP Individuals (*n* = 59) and MSAT Individuals (*n* = 60). A Mann–Whitney test in the ‘*stats*’ package was used to look for a significant difference between years, for each genetic diversity metric (Hollander and Wolfe 1973). We also calculated effective population size for SNP Individuals and MSAT Individuals using the linkage disequilibrium method in NeEstimator2 (Do et al. 2014), using only loci with allele frequencies > 0.02. Finally, pairwise relatedness (Ritland 1996) was calculated for SNP Individuals and MSAT Individuals in 2020 and 2021, using GENALEX. We assigned genetic relatedness values as follows: first order, *R*~0.5; parent–offspring or full‐sib; second order, *R*~0.25; half‐sibling, grandparent–grandoffspring, aunt/uncle–niece/nephew; third order, *R*~0.125; full cousin, great‐grandparent–great‐grandoffspring.

### 2.5 Population Abundance Analysis

Mala abundance inside the fenced reserve at Matuwa was estimated via spatially explicit capture‐recapture analyses, using the R package *secr* (Efford 2019). Separate analyses were conducted on the SNP dataset and MSAT dataset, each of which included unique individuals and recaptures. The analyses for both datasets followed the same structure. For multiple samples assigned to the same individual, the first sample collected was recorded as the initial detection, and other samples were denoted recaptures (within and between years). This detection information was combined with information about session (2020 or 2021), occasion (trapping days within a session) and detector (scat collection site) for each sample in a capture history file (capthist). The coordinates of each ‘detector’ (planned scat collection plots, including those where no scat was found) were recorded in a trap locations file (traploc), along with usage (i.e., occasions each plot was searched) and habitat covariates. The habitat covariates included four categories of vegetation cover (bare understory, dense shrubland over spinifex, scattered shrubland over spinifex, and dense mulga over tuft grass), delineated using satellite imagery as per Lohr et al. (2021). A full likelihood model, using a ‘count’ detector, and the suggested buffer width (calculated using the function suggest. buffer(); *secr*), was used to estimate density from both datasets. Each detection function (half normal, HN; hazard rate, HR; negative exponential, EN) was tested as part of a null model, and model fits were compared using Akaike Information Criterion corrected for small sample size (AICc). For both the SNP dataset and MSAT dataset, the effect of session (year), habitat, and the combined effect of session and habitat on density (*D*) and detection probability (*g*0) were tested. All models were compared using AICc and delta AIC, where delta AIC < 5 between models represented similarly well‐performing models, and the model with the lowest AICc value was deemed the best fit. Abundance was calculated as the sum product of each density estimate multiplied by the area of each habitat type within the reserve.

## 3 Results

### 3.1 SNP Panel Validation

Of the 86,871 raw ddRAD loci, 86 loci met the selection requirements for candidate SNP panel loci (Table S2). For the 86 candidate loci, a probability of identity (P_ID_) analysis showed that at least 10 SNPs were required to accurately identify unrelated individuals and 18 SNPs were required to identify siblings at the threshold of 0.0001 or exclusion probability > 99.9% as suggested by Waits et al. (2001) for SNP data (Figure S1).

Of the 50 SNP loci chosen for the panel, two loci failed to amplify in all samples during panel validation and were removed from the panel. One scat sample from 2021 had an abnormally high fail rate (amplification rate < 0.2) and was removed from further analysis. The average amplification success of the remaining samples was 0.948 ± 0.018 for tissue samples (*n* = 24 plus 3 replicates) and 0.764 ± 0.022 for scat samples (*n* = 12 plus one replicate). The rate of allelic dropout was very low for both tissue (0.0 ± 0.0) and scat (0.016 ± 0.016) replicate samples. Genotyping concordance between ddRAD and MassARRAY was 0.950 (±0.017) across 48 SNP loci in five tissue samples. Of the genotypes that did not match, almost an equal proportion of genotyping errors resulted from allelic dropout (0.021) and false alleles (0.029) sequenced on the MassARRAY.

### 3.2 Genotyping and Individual Identification

#### 3.2.1 SNP Panel

Of the 167 scat samples genotyped on the SNP panel (including the 15 replicates), 9 samples had an amplification rate < 0.2 and were removed from subsequent analyses. Additionally, 2 of the 48 loci on the panel failed to amplify across all samples, leaving 46 loci and 158 samples (including 15 replicates) for individual identification via *ScatMatch*. The average sample amplification rate was 0.757 (±0.015; range 0.25–1.0). Before calculating average allelic dropout, one technical replicate was lost due to poor amplification success (< 0.2), one was removed due to an abnormally high proportion of mismatches (0.326), which was assumed to be sample handling error, and another was removed due to abnormally low amplification success at potentially heterozygous sites (where one replicate was heterozygous at a locus, the other replicate failed to be genotyped). The average rate of allelic dropout, calculated across 12 technical replicates, was 0.045 (±0.022). When the samples were subset to include only those genotyped using both methods (microsatellites and SNPs; 116 of a possible 121 samples: 5 were dropped due to low amplification success, < 0.2), the average sample amplification rate rose to 0.805 (±0.014).

We found a significant negative association between locus error rate and amplification success (*ρ* = −0.702, *p* = 0.011), highlighting the importance of removing samples with low amplification (Figure S2). Based on the elbow graph and the frequency of allelic mismatches, we selected a sample amplification threshold of 0.7, locus amplification of 0.8 and *h* = 3 (Figure S3). This resulted in 59 unique individuals (33 unique individuals in 2020 and 26 unique individuals in 2021) identified from 87 high‐quality samples, genotyped at 30 SNP loci. We identified 28 ’recapture’ events for 16 (out of 59) individuals, within and between sampling years.

#### 3.2.2 Microsatellites

Of the total number of genotypes (*n* = 282; including both replicates of each sample), 9 (3.19%) had an amplification rate < 0.2 and were removed from further analyses, leaving 273 samples and 11 loci. For these 273 samples, the average amplification rate was 0.760 (±0.013). For the subset of samples genotyped across both microsatellites and SNPs (*n* = 242, including replicates), 6 were dropped due to a low amplification rate < 0.2, leaving 236 samples (including replicates) for which average sample amplification was very similar, 0.759 (±0.014; range 0.27–1.0). Allelic dropout was 0.081 (±0.011) and the false allele rate was 0.027 (±0.005) across the eleven loci and 118 replicate pairs. However, there was variation between PCR replicates. Average sample amplification across the first replicate for each sample was 0.674 (±0.019) and across the second replicate was 0.850 (± 0.018). Once a consensus genotype was created for each sample, amplification success for the consensus genotype was 0.917 (±0.012). At a threshold of 0.0001 (as used for SNPs), a P_ID_ analysis showed 7 loci were required to identify unrelated individuals and all 11 loci were required to identify siblings, although with less power (P_ID_ = 0.0015).

Regardless of the combination of filtering thresholds used, none of the genotype mismatch histograms showed a clear binomial distribution, nor was there a clear plateau after any of the potential mismatch thresholds. Therefore, we chose the most stringent filtering parameters (sample and locus amplification thresholds of 0.9), which resulted in 87 samples and eleven loci. Based on the elbow graphs, potentially appropriate values of *h* were 3 or 5 (Figure S4). However, there was greater overlap in the frequency distribution of within and between group mismatches for *h* = 5 (Figure S5), which represents a greater probability of misassignment due to over clustering when *h* = 5. Therefore, we used results from *h* = 3, which resulted in 60 unique individuals (37 unique individuals in 2020 and 23 unique individuals in 2021) from 87 samples, genotyped at 11 loci (i.e., all loci passed QC thresholds). We identified 27 ‘recapture’ events of 18 (out of 60) individuals within and between sampling years.

#### 3.2.3 Comparing SNP Dataset and MSAT Dataset

**FIGURE 2:**
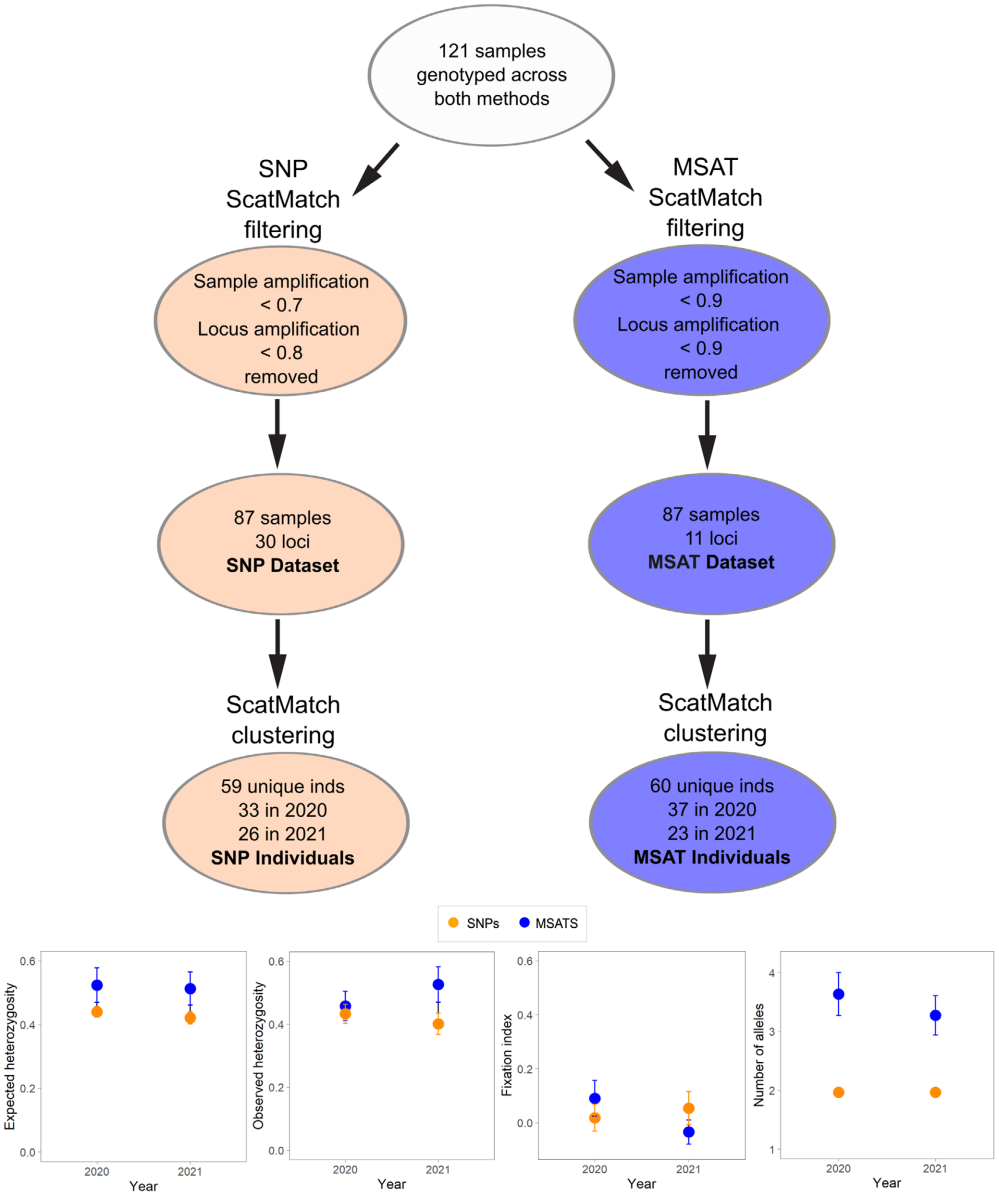
Flow chart of filtering steps involved in generating SNP and MSAT Individuals (top). Measures of genetic diversity over two years including: Expected and observed heterozygosity, fixation index and average number of alleles, calculated for SNP Individuals and MSAT Individuals (bottom). Orange points represent SNP Individuals and blue points represent MSAT Individuals. Error bars show the standard error around the mean.

Based on our chosen amplification thresholds, a similar number of samples passed for both datasets, despite more loci being lost from the SNP dataset (20 out of 50) than from the microsatellite dataset (none out of 11) (Figure 2). Average amplification was higher for the filtered microsatellite consensus genotype, although the rate of sequencing error was also higher (more than double that of the SNPs if the rates of allelic dropout and false alleles are combined). Although we had less confidence in choosing *h* for the MSAT dataset, the number of unique individuals versus recaptures (within and between years) was similar for both datasets. When we examined which samples were clustered together by both methodologies, we found that 60% of samples were assigned to the same individual by SNPs and microsatellites (Table S3). Of the mismatched samples, 34.7% were grouped by one method but were split by the other method, and 5.3% were assigned to different clusters (individuals) by the two marker types (Table S3). The extent at which samples were split or re‐grouped by SNPs or microsatellites was relatively equal and there did not appear to be any relationship between re‐grouped samples and the proportion of missing data per sample (amplification success) from SNP genotypes (χ^2^ = 0.069, *p* > 0.05) or microsatellite genotypes (χ^2^ = 0.083, *p* > 0.05), or locus error rate for SNPs (χ^2^ = 0.147, *p* > 0.05) or for microsatellites (χ^2^ = 1.90, *p* > 0.05). However, there was a significant relationship between re‐grouped samples and false allele rate (for microsatellite genotyping; χ^2^ = 5.02, *p* = 0.025), where higher false allele rates were significantly associated with samples that were grouped differently between genotyping methods.

### 3.3 Genetic Diversity and Relatedness

**FIGURE 3:**
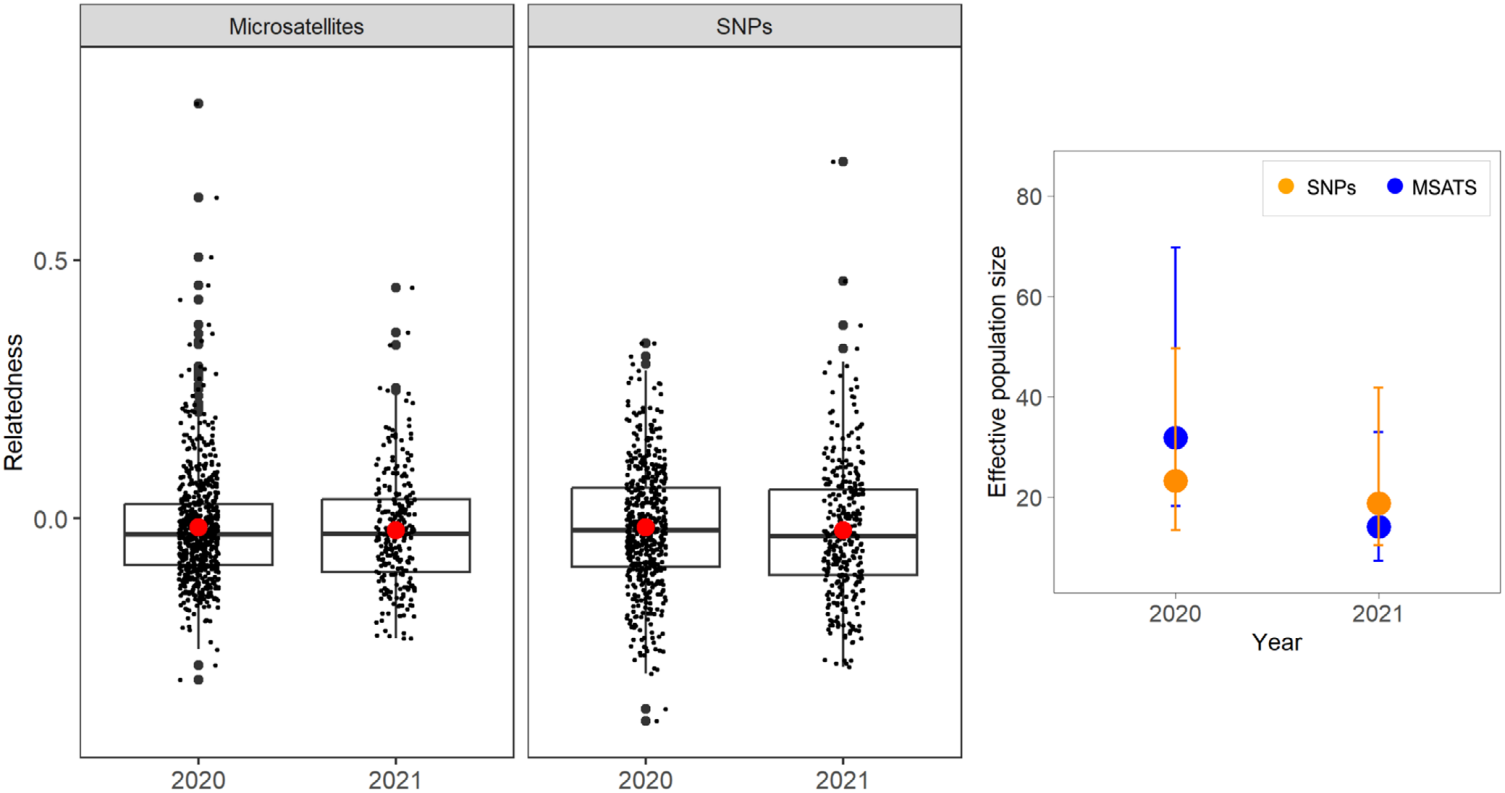
Pairwise relatedness estimates (left) calculated between MSAT Individuals and SNP Individuals. Red points represent the mean of each group. On the right, effective population size estimated for SNP Individuals (orange points) and MSAT Individuals (blue points). Error bars represent the 95% confidence limits of each estimate.

For both SNP Individuals and MSAT Individuals, we found no evidence of non‐random mating that could suggest inbreeding. The inbreeding coefficient was close to zero across time for both datasets, and estimates of observed and expected heterozygosity were similar, changing slightly over time but not significantly (Figure 2; *p* > 0.05 in all comparisons). When expected heterozygosity was calculated for the SNP Individuals and MSAT Individuals, a slight decline over time was evident across both datasets (Figure 2; *p* > 0.05). In contrast, for MSAT Individuals, there was an increase in observed heterozygosity over time, but a decrease in observed heterozygosity was evident from the SNP Individuals (Figure 2; *p* > 0.05). Since the genotyping methods were different, the values on the y‐axis are not comparable; rather, it is the trend over time that is noteworthy. For the SNP Individuals, Wright's fixation index and the average number of alleles showed little to no change over time, whereas MSAT Individuals showed a declining trend for both measures (Figure 2). However, none of the genetic diversity metrics changed significantly from 2020 to 2021 (*p* > 0.05 for all comparisons). Both datasets showed low effective population size across years (Figure 3; right panel). The estimated effective population of mala from SNP Individuals was 23.3 (13.5–49.6) in 2020 and 18.8 (10.5–41.8) in 2021, and from MSAT Individuals was 31.9 (18.3–69.7) in 2020 and 14.1 (7.3–33.0) in 2021 (Figure 3).

The range of pairwise relatedness estimates was greater for MSAT Individuals than for SNP Individuals (Figure 3), and there was little overlap in which samples were identified to be related (*R* > 0.125) between the two datasets. However, mean pairwise relatedness was similar for individuals from both datasets and across years (Figure 3; left panel), indicating a low proportion of related individuals. For SNP Individuals, we detected 16.9% of pairwise comparisons in 2020 and 12.1% in 2021 with first to third order pedigree relationships (*R* > 0.125). We found a slightly lower proportion of related individuals with the same pedigree relationships among MSAT Individuals (8.6% in 2020 and 9.5% in 2021).

### 3.4 Population Abundance

**FIGURE 4:**
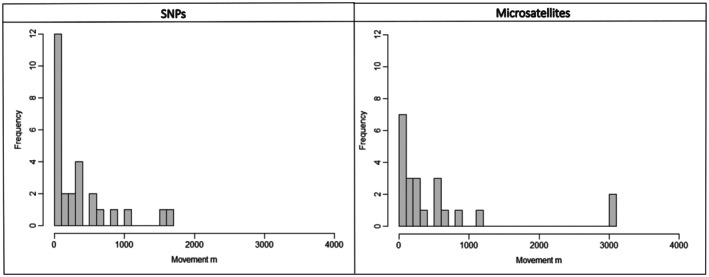
Frequency of distance between recorded mala recaptures in metres, using individual identification data obtained from the SNP dataset (left) and MSAT dataset (right).

For the SNP dataset, we identified a total of 87 detections of 59 unique individuals (28 recaptures, including detections within and between years; identified via *ScatMatch*), that were suitable for use in the SECR model. Similarly, we identified 87 detections using the MSAT dataset, including 60 unique individuals and 27 recaptures. For the SNP dataset, the average distance between recaptures was 312.8 m (0–1600 m). Of the 28 recaptures of 16 individuals, 51.9% were collected less than 200 m apart (and 13 of those were from the same plot i.e., within the 5 m radius of other samples) and 19% were collected over 600 m apart (Figure 4). Of 27 recaptures of 18 individuals identified in the MSAT dataset, 43.5% of recaptures were within 200 m, 21.7% were collected more than 600 m apart and one sample was collected 3013.6 m apart (Figure 4). The hazard detection function was the best fit for models from both datasets (Table S4).

The best performing multisession SECR model for both datasets was when density varied with session and habitat, while all other variables remained constant (Tables 1, 2). For the MSAT dataset, three similar models performed well (dAICc < 3.64; Table 2). However, the models ranked second and third resulted in standard error estimates that were greater than the density estimates for some habitat categories, suggesting weakness in model parametrization. Therefore, we have used the results from the model ranked first (Table 2), rather than averaging across the three top ranked models.

**TABLE 1:** AIC comparison of models fitted for a closed population, multisession SECR model for mala density, using detection information from the SNP dataset. Parameters in these models relate to density (*D*), capture probability (g_0_), sigma (σ), shape (z), and habitat.

Rank	Model	Detection function	Parameters	dAICc
1	*D* ~ habitat + session, g_0_ ~1, σ ~1, z ~1	Hazard rate	8	0.00
2	*D* ~ habitat, g_0_ ~1, σ ~1, z ~1	Hazard rate	7	0.01
3	*D* ~1, g_0_ ~ habitat + σ ~1, z ~1	Hazard rate	7	6.50
4	*D* ~ session, g_0_ ~1, σ ~1, z ~1	Hazard rate	5	6.59
5	*D* ~1, g_0_ ~ habitat + session, σ ~1, z ~1	Hazard rate	8	6.66
6	*D* ~1, g_0_ ~ session, σ ~1, z ~1	Hazard rate	5	6.77
7	*D* ~1, g_0_ ~1, σ ~1, z ~1	Hazard rate	4	6.86
8	*D* ~ habitat + session, g_0_ ~ habitat + session, σ ~1, z ~1	Hazard rate	12	10.08

**TABLE 2:** AIC comparison of models fitted for a closed population, multisession SECR model for mala density, using detection information from the MSAT dataset. Parameters in these models relate to density (*D*), capture probability (g_0_), sigma (σ), shape (*z*), and session or habitat.

Rank	Model	Detection function	Parameters	dAICc
1	*D* ~ habitat + session, g_0_ ~1, σ ~1, *z* ~1	Hazard rate	8	0.00
2	*D* ~ habitat + session, *g*_0_ ~ habitat + session, σ ~1, *z* ~1	Hazard rate	12	2.26
3	*D* ~ habitat, *g*_0_ ~1, σ ~1, *z* ~1	Hazard rate	7	3.64
4	*D* ~ session, *g*_0_ ~1, σ ~1, *z* ~1	Hazard rate	5	11.64
5	*D* ~1, *g*_0_ ~ habitat, σ ~1, *z* ~1	Hazard rate	7	12.88
6	*D* ~1, *g*_0_ ~ habitat + session, σ ~1, *z* ~1	Hazard rate	8	13.50
7	*D* ~1, *g*_0_ ~1, σ ~1, *z* ~1	Hazard rate	4	15.28
8	*D* ~1, *g*_0_ ~ session, σ ~1, *z* ~1	Hazard rate	5	15.75

**FIGURE 5:**
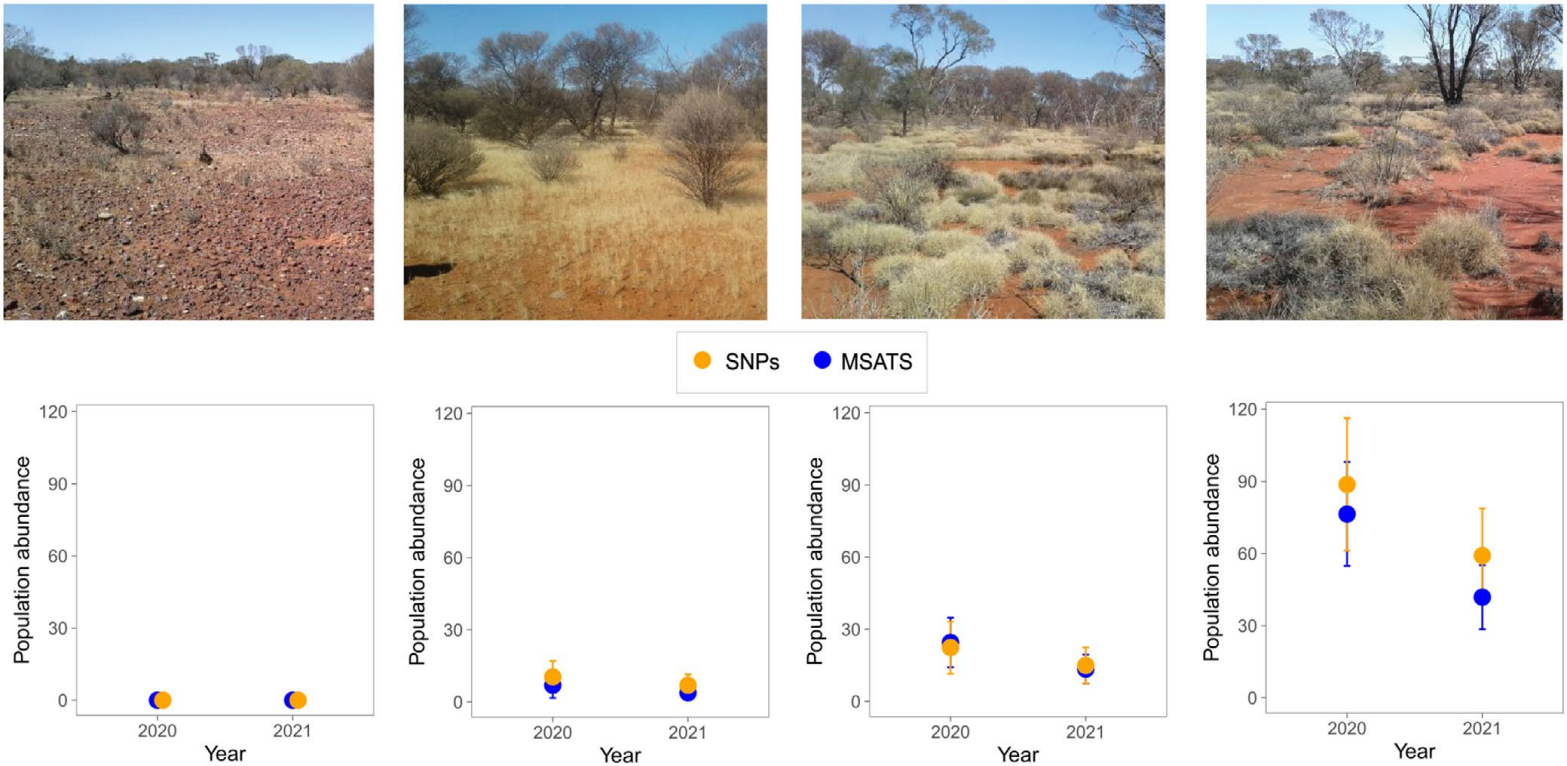
Mala population abundance estimates from *secr* analyses across two years and four different habitat categories, including (from left) bare understory, dense mulga over tuft grass, dense shrubland over spinifex and scattered shrubland over spinifex. Orange represents estimates derived from the SNP dataset and blue represents estimates derived from the MSAT dataset.

Mala abundance decreased dramatically from 2020 to 2021, and this pattern was consistent across both datasets (Figure 5, Tables S5 and S6). Based on the SNP dataset, there were approximately 122 (±45) mala in the reserve in 2020 and 81 (±32) in 2021. Similarly, based on the MSAT dataset, there were approximately 108 (±37) mala in 2020 and 59 (±22) in 2021. Abundance also varied across habitat type, with effectively no mala inhabiting bare understory, few found in dense mulga with tuft grass (about 8.5% of individuals based on the SNP panel and about 6% based on microsatellites), about 20% in dense shrubland over spinifex and approximately 70% occupying scattered shrubland over spinifex (Figure 5).

## 4 Discussion

Long‐term monitoring of translocated fauna to fenced reserves is critical to achieve continued translocation ‘success’ and avoid undocumented reasons for translocation failure. However, accurate and efficient population monitoring is notoriously difficult for cryptic, trap avoidant species, highlighting the importance of continually developing techniques to accurately identify individuals from non‐invasively collected samples to estimate population abundance. In this study, we document the design and validation of a species‐specific SNP panel, developed for the identification of individual mala from scat DNA, and subsequently to estimate mala abundance over time and across habitat types. We compare the genotyping success of the SNP panel to an existing microsatellite‐based methodology (Treloar et al. 2023) and explore the effect of genotyping quality on the accuracy of individual identification and abundance estimates using both methods. Counter to expectation, the SNP panel did not have higher amplification success than the microsatellites, but genotyping error rates were reduced as anticipated. The number of unique individuals identified using the SNP panel was very similar to the number identified using microsatellites, and the consistency between the two datasets carried through to our estimates of population abundance. However, there were mismatches between the two genotyping methods as to which samples were identified as unique individuals. There was no obvious directional pattern of over splitting or over clustering based on either marker; however, high microsatellite false allele rates were significantly associated with mismatched samples.

Typically, the amplification success of scat DNA samples genotyped on a SNP panel is greater than the genotyping success of microsatellites, when the two methods have been directly compared (Ekblom et al. 2021; von Thaden et al. 2017). We found equivocal evidence of such an effect in our study, with individual microsatellite genotyping runs having amplification success rates of 67% and 85%, compared to 81% for the SNP panel. However, employing the ‘multi‐tubes’ approach for microsatellite genotyping enabled us to generate consensus genotypes for a final success rate of 91.7%. Potentially, a higher success rate could have been achieved for the SNP dataset if we had taken the same genotyping approach as for the microsatellites, but using a multi‐tubes approach adds substantially to project costs (Ekblom et al. 2021), and we found that there was high consistency in our replicate samples (genotyping error rate 4.5%) indicating there may not have been much gain from doing so. Further, we may have detected variation in amplification success between the two methods as about 60% of samples genotyped on the SNP panel were DNA extracts from replicate scats, rather than the same DNA extract used for microsatellite genotyping (insufficient DNA was available for some samples), complicating direct comparisons. Additionally, mala scat is collected from open habitat in the remote arid zone where scat is exposed to high temperatures and UV, and mala have a predominantly herbivorous diet, all of which are factors that serve to reduce the DNA quality and quantity extracted from scat (Cowen et al. 2022; Ramón‐Laca et al. 2015). It's possible that the two genotyping approaches differ in their sensitivity to these factors. Nevertheless, the SNP panel outperformed the microsatellites with regard to reduced error rates, highlighting the greater genotyping precision of the SNP panel compared to microsatellites (Carroll et al. 2018; Fabbri et al. 2012; Kraus et al. 2015; von Thaden et al. 2017). Lower error rates were likely due to the use of allele‐specific primers and automated SNP calling, which reduces the opportunity for human error (Thavornkanlapachai et al. 2024). In contrast, microsatellite allele calling is influenced by interpretation of electropherograms (Fabbri et al. 2012), where primer‐dimer peaks and stutter bands impact allele assignment (Guichoux et al. 2011; Kraus et al. 2015), contributing to mistyping.

Higher precision in SNP genotyping provided more confidence in our individual classifications using this method, a finding also supported by Ekblom et al. (2021). However, given that we do not have associated tissue samples from the known population, the accuracy of our individual classifications cannot be definitively assessed. We noted a greater distinction between within‐individual (i.e., samples collected from the same individual) genotyping differences and between‐individual genotyping differences based on SNP genotyping when assessing the optimal allelic mismatch threshold (*h*) for each dataset, enabling us to have higher confidence in our choice of *h* and the resultant sample clustering. Even though a similar number of unique individuals (and recaptures) were identified using both genotyping methods, multiple mismatches in the assignment of scats to individuals highlighted potential misassignment of samples in one or both datasets. The lack of a (consistent) pattern of clustering or splitting of scats across groups by either genotyping method makes it difficult to interpret the reason for individual assignment discrepancies. However, genotyping errors likely contributed to re‐grouping in some cases, particularly the higher false allele rate of microsatellites, as evident in this study among others (Fabbri et al. 2012; von Thaden et al. 2017). Higher genotyping errors would contribute to over‐splitting of samples into (falsely) distinct individuals. Further, other studies have noted the reduced statistical power of microsatellites compared to SNP panels (Thavornkanlapachai et al. 2024) which results in reduced capability to discriminate between scats from different individuals, leading to over‐merging. Our dataset comprised 40 alleles for the microsatellite dataset versus 59 alleles for the SNP dataset, indicating less discriminatory power in the microsatellite dataset, supporting this observation.

Discrepancies in the assignment of scats to individuals between the microsatellites and SNPs transpired as spatial differences between recaptures, which may have implications for spatial mark‐recapture analyses based on these data and assessment of species' home ranges. Previous data suggest mala have a home range of approximately 7.96 ha in Shark Bay, Western Australia (Hardman 2006), and 11.35 ha at Uluru—Kata Tjuta National Park (Clayton 2012), which equate to a radius of 160 to 190 m. Individual classifications based on the SNP dataset were more consistent with these observations. For example, 52% of individuals identified by SNPs in this study were collected within 200 m of each other, with a maximum distance of 1600 m between samples from one individual; whereas only 43.5% of individuals' scats identified by microsatellites were within 200 m of each other, 21.7% were more than 600 m apart, and samples from one individual were collected 3013 m apart. The results of the SNP individual assignment are more consistent with our knowledge of mala ecology, further supporting our conclusion that SNP genotypes more accurately assign scats to individuals.

Despite discrepancies in individual assignment, overall, genetic diversity patterns, effective population size, and estimates of mean pairwise genetic relatedness were similar regardless of genotyping method, increasing our confidence in detecting trends within the Matuwa mala population. Expected heterozygosity was consistent and we found low to no evidence of non‐random mating (e.g., inbreeding). Of note, however, is that our SNP panel genetic diversity estimates are less sensitive to change as a result of the marker selection process, where the genotyped SNPs are biased towards high minor allele frequency (MAF) and high heterozygosity to maximise information content for individual identification. Loci biased towards high MAF are relatively insensitive to diversity changes, in contrast to microsatellites, which have more rare alleles, making them more sensitive to population genetic changes through time (Geibel et al. 2021). Consistent with this, we observed a greater magnitude of change across years in genetic metrics that are more sensitive to rare alleles (number of alleles and effective population size) in the microsatellite dataset than the SNP dataset. Future genetic monitoring of this population would require parameter estimation with a broader, unbiased set of SNP markers to ensure estimates are representative and sensitive enough to detect among year variation.

The limited evidence of inbreeding in the Matuwa population was surprising given the multiple sequential founder effects that could be caused by multiple translocations (Lambert et al. 2005), and the increased risk of inbreeding within small, isolated and fenced populations (Hayward and Kerley 2009; Ottewell et al. 2014). Such limited evidence of inbreeding could be due to admixture between the three source populations used to establish the Matuwa mala population (Figure 1). There are multiple examples of the benefits of intraspecific admixture for conservation purposes, specifically to improve genetic diversity and reduce the potential for inbreeding depression in the mixed population (Frankham 2015; Nistelberger et al. 2023; Semple et al. 2020; White et al. 2018). However, given the decline in mala abundance from 2020 to 2021 and the low effective population size evident in both years, we may find evidence of inbreeding in subsequent generations.

The consistency in the number of individuals identified and the estimates of species density across the two different genotyping techniques gives us high confidence that the decline in the abundance of mala in the fenced area on Matuwa is real. Until now, the Matuwa mala population has been considered stable (Lohr 2019; Sims et al. 2017), and the last estimate of their abundance showed a substantial (~70%) increase in mala numbers since the final translocation to Matuwa in 2013 (Treloar et al. 2023). However, we found evidence of a 40% decline in abundance from 2020 to 2021. This decline was likely due to a period of prolonged drought from 2018 to 2020 (Australian Bureau of Meteorology 2020). Furthermore, 2019 was the driest year on record for Matuwa (annual rainfall, 69.5 mm; mean annual rainfall, 260.8 mm; Australian Bureau of Meteorology 2021). Drought conditions can negatively impact macropods by reducing local vegetative productivity, which limits food and habitat availability (Lundie‐Jenkins 1993a), subsequently reducing body condition (Stirrat 2003); disrupting breeding (Lundie‐Jenkins 1989) and increasing juvenile mortality (Fisher et al. 2001). Historical accounts of mala population fluctuations have been associated with drought conditions (Finlayson 1961; Richards 2012), and ultimately attributed to reduced local productivity of vegetation due to low rainfall, and changes in habitat structure related to time since fire (Lundie‐Jenkins, 1993).

A similar population trend was seen in the boodie population in the Matuwa reserve, which experienced a substantial decline in 2019, associated with drought conditions and browsing damage due to their abundance before the crash (Treloar et al. 2021). In response to the decline in boodie numbers, we provided supplemental food and water to the semi‐wild populations of marsupials between August 2019 and September 2020. Preliminary analysis of camera‐trap data, collected during daily monitoring for incursions of introduced predators into the reserve, suggests that the boodie population dominated the use of the supplemental resources and subsequently boomed in abundance (unpublished data). Further, boodies exhibit aggressive behaviour towards conspecifics and other species around supplemental food resources (unpublished data).

Boodie population dynamics (from 2018–2020), and the provisioning of supplemental resources likely exacerbated the negative effects of drought conditions on the mala population. Despite there being evidence of resource partitioning between boodies and mala (Lush et al. 2017; Sato et al. 2018), the two species consume a similar diversity of plant and fungal species, but at different volumes, with different focal taxa (Treloar 2021). Therefore, during periods of extended drought, when resource limitation extends across multiple years, interspecific competition between the two species likely intensifies. Particularly, as the boodie population increased in response to supplemental food and water, their competitive impact on the mala, and other sympatric species likely intensified. Therefore, increased competition from a rapidly recovering population of boodies may have contributed towards the continued decline in mala in 2020 and 2021.

In addition to providing a means to estimate population abundance, scat monitoring allows detection of habitat preferences in cryptic target fauna. Another prominent pattern that was supported by both datasets (SNP panel and microsatellites) was that mala were predominantly found in scattered shrubland over spinifex, similar to the habitat preference shown by golden bandicoots (Lohr et al. 2021). Spinifex provides an important source of food and shelter, providing insulation from the extreme temperature variation of the arid zone (Chambers and Dickman 2002; Kinlaw 1999; Lohr et al. 2021). Specifically for mala, variation in the density of spinifex coverage is important, with dense spinifex providing more shelter from the elements and predators, while less dense spinifex habitat provides a greater diversity and abundance of food species (Lundie‐Jenkins 1993a). Open mulga ( *Acacia aneura* ) woodlands with tufted grasses and bare ground are two habitat types where few to no mala were found, likely because they do not provide adequate shelter or dietary resources for mala.

### 4.1 Management Implications

Further surveys of the mala population in the Matuwa reserve are required to determine if the population recovered following high rainfall events in 2022 and 2023, and follow up genetic assessments could help to determine any evident genetic impacts of the decline (e.g., increased inbreeding, loss of genetic diversity). Mala are an endangered species and effectively extinct in the wild since they only occur in four semi‐wild fenced areas and on one offshore island, with extant populations already having been exposed to multiple bottlenecks. Therefore, it is critical to manage the demographic and genetic health of these semi‐wild, reintroduced populations to ensure the species' future viability and ongoing recovery. Further, additional improvements could be made to our non‐invasive sampling approach to improve the cost‐effectiveness and accuracy of the monitoring method, which is especially important when mala are released into larger fenced areas or co‐occur with species that dominate traps, which reduces the effectiveness of traditional monitoring approaches (live trapping, cameras). Whilst our mark‐recapture estimates provided plausible estimates of population abundance, the error bounds on these estimates were large, likely due to the relatively small sample size used to estimate abundance as many samples were lost due to sequencing failure or low genotyping success, reducing the power of our analysis. Future surveys should consider including more scat collection points (“capture sites”) along each transect and include multiple capture occasions at each site. This should have the effect of decreasing the ratio of unique individuals to the number of recaptures which will reduce the standard error of density estimates and hence improve their precision (Schmidt et al. 2022). Although we achieved decent genotyping success rates for scat DNA, genotyping success could be further improved by modifying field collection protocols to reduce the amount of time that scat is exposed to UV radiation, for example, aiming to collect fresh scat shortly after sunrise and/or selecting samples which are shaded or partially buried to maximise DNA retention, although this may be difficult to achieve given the species' preference for open (exposed) habitat types. Alternatively, scat DNA extracts could be screened for the presence and quantity of mala DNA prior to genotyping to reduce genotyping costs, using quantitative PCR (qPCR). For example, gBlock screening involves the use of species‐specific primers and a piece of artificial DNA that, when PCR amplified, can be used to determine how much mala DNA is present in each sample (Conte et al. 2018).

## Author Contributions

**Deanne Cummins:** conceptualization (supporting), formal analysis (lead), methodology (equal), project administration (lead), writing – original draft (lead), writing – review and editing (equal). **Kristen Nilsson:** formal analysis (supporting), methodology (equal), writing – review and editing (equal). **Rujiporn Thavornkanlapachai:** conceptualization (equal), formal analysis (supporting), methodology (supporting), resources (equal), validation (equal), writing – review and editing (equal). **Kym Ottewell:** conceptualization (equal), data curation (equal), formal analysis (supporting), funding acquisition (equal), methodology (equal), resources (equal), validation (equal), writing – review and editing (equal). **Cheryl Lohr:** conceptualization (equal), data curation (equal), formal analysis (supporting), funding acquisition (lead), methodology (supporting), project administration (equal), resources (equal), writing – review and editing (equal).

## Funding

This work was supported by the Chevron Gorgon Barrow Island Threatened and Priority Species Translocation Program.

## Conflicts of Interest

The authors declare no conflicts of interest.

## Supporting information


**Table S1:** History of conservation introductions and translocations of mala ( *Lagorchestes hirsutus* ‘central subspecies’) in Australia.
**Table S2:** Filtering parameters used to obtain candidate SNPs for the MassARRAY SNP panel for individual identification.
**Figure S1:** The probability of identity between unrelated (green line; PID) and related individuals (orange line; PIDsib), considering all candidate loci for the mala SNP panel.
**Figure S2:** Locus error rate calculated using replicate pairs of SNPs (above) and microsatellites (below), plotted against the amplification success of each respective genotyping method.
**Figure S3:** Number of groups of unique individuals at each mismatch threshold ('*h*'; left column) and the frequency of each number of mismatches (right column), based on the SNP dataset, generated using '*ScatMatch*'. Each row represents a different combination of filtering parameters.
**Figure S4:** Number of groups of unique individuals at each mismatch threshold ('*h*'; left column) and the frequency of each number of mismatches (right column), based on the microsatellite dataset, generated using '*ScatMatch*'. Each row represents a different combination of filtering parameters.
**Figure S5:** Misassignment graphs for different microsatellite mismatch thresholds generated in '*ScatMatch*' (left *h* = 3 and right *h* = 5) showing the frequency of allelic mismatches where both samples have genotypes. The red dashed line represents the upper 0.995 percentile of pairwise mismatches ‘within’ groups and the blue dashed line represents the lower 0.005 percentile of mismatches ‘between’ groups.
**Table S3:** Assignment of scat DNA samples to unique individuals via '*ScatMatch*', based on SNP panel versus microsatellite genotyping.
**Table S4:** AIC comparison of null models fitted for closed population, multisession SECR model for mala density, using individual identification data obtained from SNP dataset and MSAT dataset. Parameters in these models relate to density (D), capture probability (g0), sigma (σ), and shape (z).
**Table S5:** Mala density (*D*) and abundance for each session (year) and habitat type, based on the SNP dataset.
**Table S6:** Mala density (*D*) and abundance for each session (year) and habitat type, based on the MSAT dataset.

**Data S1:** Supplementary file 2

**Data S2:** Supplementary file 3

**Data S3:** Supplementary file 4—Mala_SNPpanel_ID_sequences

## Data Availability

Sequences for microsatellite marker alleles are available on the NCBI database (ON184275‐ ON184277, ON184278‐ ON184280, ON184282‐ ON184283, ON184284‐ ON184286). Raw ddRAD reads from which the SNP panel was designed are available online from the Oz Mammals Genomics Initiative data portal (https://data.bioplatforms.com/dataset; dataset IDs: 102.100.100/52587 and 102.100.100/52587). The metadata for scat samples used in the study are attached as file S2. The code used for all analyses is attached as file S3. The SNP panel loci are attached as Supplementary file S4.
