# Supplementary material for: Estimating the Abundance of a Cryptic, Endangered Marsupial, Mala ( Lagorchestes hirsutus ) Using Microsatellite and Single Nucleotide Polymorphism Genotyping Panels
Source: Ecol Evol. 2026 Apr 29;16(5):e73480. doi: 10.1002/ece3.73480 (PMC13125950; doi:10.1002/ece3.73480)
Supplement: Supplementary file 1 — Table S1: History of conservation introductions and translocations of mala ( Lagorchestes hirsutus ‘central subspecies’) in Australia. Table S2: Filtering parameters used to obtain candidate SNPs for the MassARRAY SNP panel for individual identification. Figure S1: The probability of identity between unrelated (green line; PID) and related individuals (orange line; PIDsib), considering all candidate loci for the mala SNP panel. Figure S2: Locus error rate calculated using replicate pairs of SNPs (above) and microsatellites (below), plotted against the amplification success of each respective genotyping method. Figure S3: Number of groups of unique individuals at each mismatch threshold ('h'; left column) and the frequency of each number of mismatches (right column), based on the SNP dataset, generated using 'ScatMatch'. Each row represents a different combination of filtering parameters. Figure S4: Number of groups of unique individuals at each mismatch threshold ('h'; left column) and the frequency of each number of mismatches (right column), based on the microsatellite dataset, generated using 'ScatMatch'. Each row represents a different combination of filtering parameters. Figure S5: Misassignment graphs for different microsatellite mismatch thresholds generated in 'ScatMatch' (left h = 3 and right h = 5) showing the frequency of allelic mismatches where both samples have genotypes. The red dashed line represents the upper 0.995 percentile of pairwise mismatches ‘within’ groups and the blue dashed line represents the lower 0.005 percentile of mismatches ‘between’ groups. Table S3: Assignment of scat DNA samples to unique individuals via 'ScatMatch', based on SNP panel versus microsatellite genotyping. Table S4: AIC comparison of null models fitted for closed population, multisession SECR model for mala density, using individual identification data obtained from SNP dataset and MSAT dataset. Parameters in these models relate to density (D), capture probability (g0 [file ECE3-16-e73480-s001.docx]

**Supplementary material**

**Estimating the abundance of a cryptic, endangered marsupial, mala (*Lagorchestes hirsutus*) using microsatellite and Single Nucleotide Polymorphism genotyping panels**

Deanne Cummins ^1^, Kristen Nilsson^1^, Rujiporn Thavornkanlapachai^1^, Kym Ottewell^1^, and Cheryl Lohr ^1^

Table S1: History of conservation introductions and translocations of mala (*Lagorchestes hirsutus* ‘central subspecies’) in Australia.

| **Source Population** | **Release Population** | **Code** | **History** | **Number founders** | **Year** | **Citation** |
| --- | --- | --- | --- | --- | --- | --- |
|  |  |  |  |  |  |  |
| Tanami Desert, Northern Territory, Extinct in wild. | Arid Zone Research Institute, captive | AZRI | The last 2 wild colonies on Tanami Hwy in Northern Territory, 15km apart, 450 km NW of Alice Springs, and 10 km from Sangsters Bore with an estimated total of 50-100 individuals. A total of 22 founders taken from the wild over 6 years. Sangster’s Bore colony confirmed extinct in 1987 due to fox predation. Tanami Hwy population became extinct in 1991 due to wild fire. | 5 (1M: 4F)  22 | 1981  1986 | (Bolton & Latz, 1978; Johnson et al., 1996; Langford & Burbidge, 2001) |
| Arid Zone Research Institute | Release to wild near Lake Surprise, NT, and Sangster’s Bore, NT |  | Experimental releases of captive bred animals back into the wild. Ultimately, all releases failed, leading to creation of mala paddock. | 12  13  81  51 | 1984  1985  1990-1992  1989-1993 | (Johnson et al., 1996; Richards, 2012) |
| Arid Zone Research Institute | ‘Mala Paddock’, Lander River | MALP | Fenced refuge, 1 km^2^. First release occurred in 1986 with some mala escaping the fenced refuge. More fencing material was added, but dingoes entered the enclosure. Multiple releases between January and May of 1987. The population increased to approximately 150 - 200 individuals by 1992. Mala paddock discontinued in 2001. | 12  47 (20M: 27F) | 1986  1987 | (Clayton, 2012; Friend & Beecham, 2004; Johnson et al., 1996; Langford & Burbidge, 2001) |
| Arid Zone Research Institute | Western Plains Zoo |  | The program was discontinued in 2001. | ? | 1988 | (Clayton, 2012; Richards, 2012) |
| Arid Zone Research Institute | Monarto Zoological Park |  | The program was discontinued in 2004. | ? | 1992-1995 | (Clayton, 2012; Richards, 2012) |
| ‘Mala Paddock’, Lander River | Tanami Desert, Northern Territory |  | Attempt to return to wild at Yinapaka release site (1990 and 1991 releases) and Lungkartajarra release site (series of releases 1989-1991). A further 81 released immediately outside the ‘Mala Paddock’ between 1990 – 1992. Failed due to feral cat (*Felis catus*) and red fox (*Vulpes vulpes*) predation. Some releases persisted for 20-38 months. | 11  20 (8M: 12F)  23  81 | 1990  1991  1989 - 1991  1990-1992 | (Gibson et al., 1994; Hardman, 2006; Langford & Burbidge, 2001) |
| Arid Zone Research Institute | Alice Springs Desert Park | ASDP | Captive, two fenced areas (0.04 km^2^ and 0.50 km^2^), established originally from captive bred population in AZRI with supplemental animals from other sources. | 53 | 1997 | (Nally et al., in draft; Richards, 2012) |
| Western Plains Zoo |  |  |  | 9 |  |  |
| Scotia Sanctuary |  |  |  | 3 | 2020 |  |
| ‘Mala Paddock’, Lander River |  |  |  | 1 |  |  |
| Watarrka National Park |  |  |  | 1 |  |  |
| Uluru-Kata Tjuta National Park |  |  |  | 2 |  |  |
| Mala Paddock | Return to Dryandra captive breeding facility | RTDR | Captive, two 10 ha breeding enclosures, established from semi-wild ‘Mala paddock’ in 1998. Operational until 2012. | 19 | 1998 | (Friend & Beecham, 2004) |
| Mala Paddock | Peron Captive Breeding Centre, Shark Bay, WA | PCBC | Captive, 1.5 ha breeding enclosures established from Mala Paddock population in 1998, operational until 2011. | 29 (11M: 18F) | 1999 | (Morris et al., 2004) |
| Peron Captive Breeding Centre | Francois Peron National Park, WA |  | 1050 km^2^ fenced peninsula with predator control. Release ultimately failed due to feral cat predation. | 16 (9M: 7F) | 2001 | (Morris et al., 2004) |
| Mala Paddock | Trimouille Island, WA | TRIM | Offshore island haven, 5.2 km^2^. 30 adults, 20 females, 11 with pouch young. | 30 (10M: 20F) | 1998 | (Langford & Burbidge, 2001) |
| Mala Paddock | Watarrka National Park | WATA | Fenced refuge, 120 ha. Mala paddock discontinued in 2001. | 96 | 2000-2001 | (Clayton, 2012; Langford & Burbidge, 2001; Richards, 2012) |
| Trimouille Island | Matuwa Kurrara Kurrara National Park | MATU | Fenced refuge, 11 km^2^. Extant. | 34 | 2011 | (Nally et al., in draft) |
| Peron Captive Breeding Centre |  |  |  | 25 |  |  |
| Return to Dryandra captive breeding facility |  |  |  | 12 |  |  |
| Alice Springs Desert Park | Scotia Sanctuary, Australian Wildlife Conservancy, NSW | SCOT | Fenced refuge, 650 km^2^. Removed 2020. | 6  19 | 2001  2004 | (Richards, 2012) |
| Watarrka National Park | New Haven, Australian Wildlife Conservancy, NT | NEHA | Fenced refuge, 94 km^2^. | 27 | 2017 | [Newhaven - AWC - Australian Wildlife Conservancy](https://www.australianwildlife.org/where-we-work/newhaven/), (Nally et al., in draft) |
| Scotia Sanctuary |  |  |  | 34  42 | 2018  2020 |  |
| Alice Springs Desert Park |  |  |  | 9 | 2019 |  |
| Watarrka National Park | Uluru-Kata Tjuta National Park |  | Fenced refuge, 1.7 km^2^. Extant. | 24 | 2005 | (Clayton, 2012; Nally et al., in draft; Richards, 2012) |

Table S2: Filtering parameters used to obtain candidate SNPs for the MassARRAY SNP panel for individual identification.

| Filtering parameters | Minimum | Maximum | Number loci |
| --- | --- | --- | --- |
| Individual read depth | 5 | NA | 86809 |
| Remove duplicate SNPs | NA | NA | 35396 |
| SNP position | 30 | 110 | 21131 |
| Genotyping quality | 30 | NA | 21130 |
| Average locus read depth | 8 | 100 | 20474 |
| Maximum read depth | NA | 100 | 20432 |
| Locus call rate | 0.78 | NA | 3236 |
| Individual call rate | 0.8 | NA | 73 (Inds) |
| Paralogs removed | 0.2 | NA | 3228 |
| MAF | 0.34 (focal);  0.14 (other) | NA | 303 |
| Heterozygosity | 0.4 | 0.6 | 265 |
| HWE | 0.05 | NA | 233 |
| Allele balance | 0.44 | 0.56 | 231 |
| Linkage disequilibrium | NA | 0.3 | 95 |
| SNPs on the same scaffold | 1000bp | NA | 86 |


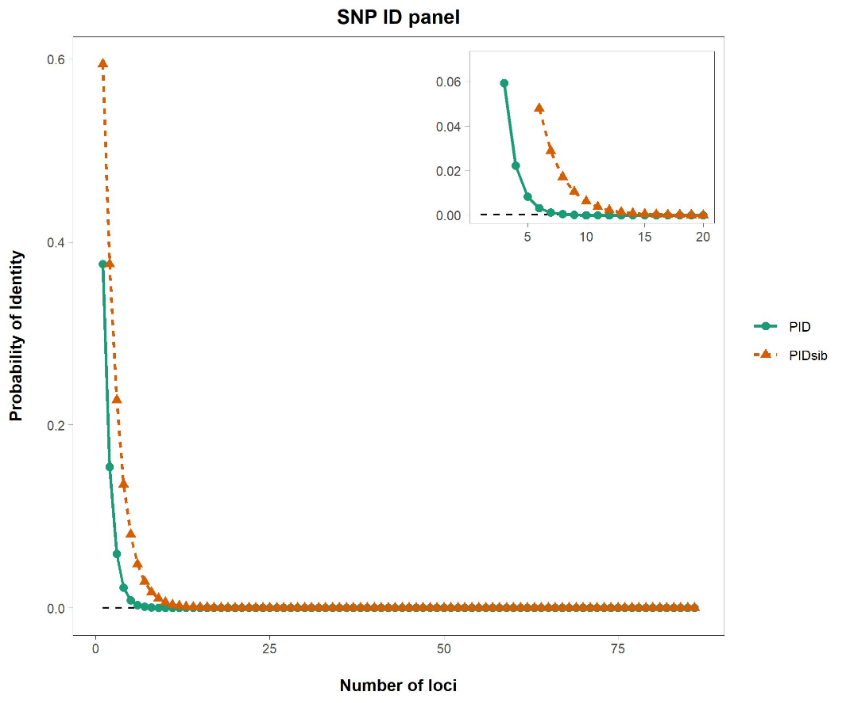


Figure S1: The probability of identity between unrelated (green line; PID) and related individuals (orange line; PIDsib), considering all candidate loci for the mala SNP panel.


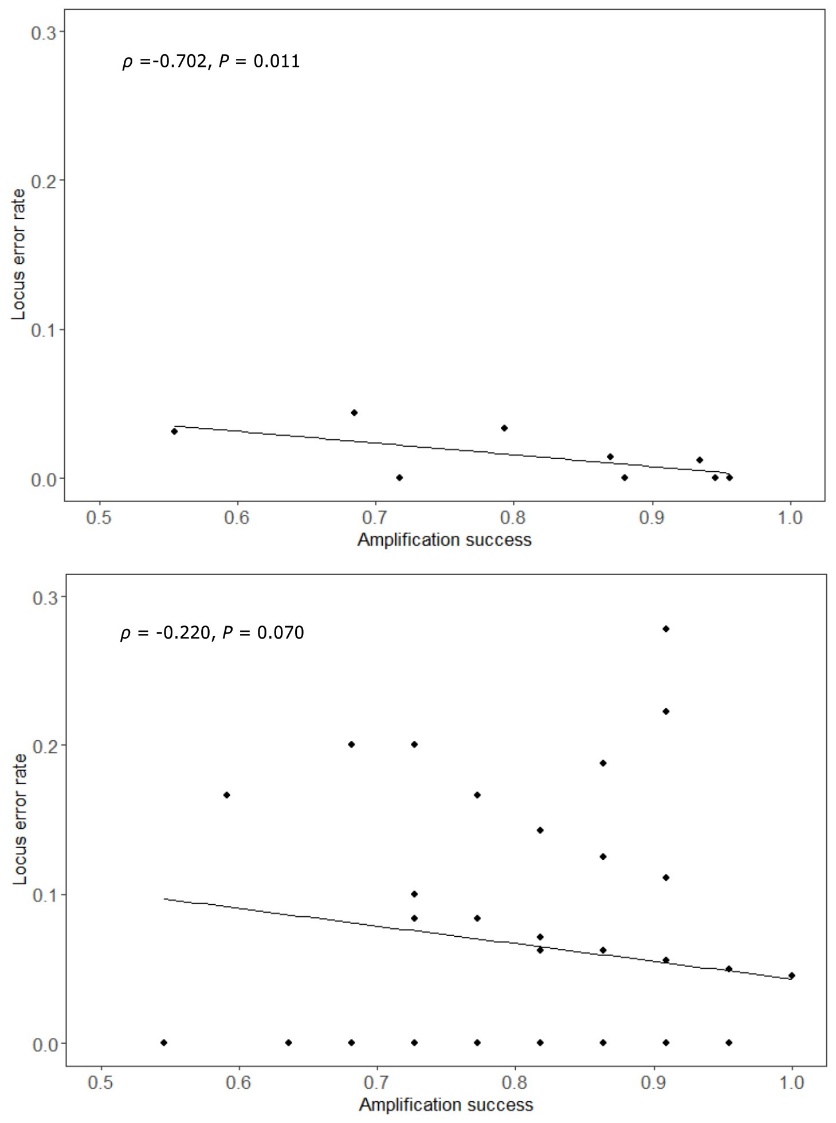


Figure S2: Locus error rate calculated using replicate pairs of SNPs genotyped on the mala SNP panel (top panel), and microsatellites (bottom panel), plotted against the amplification success of each respective genotyping method.


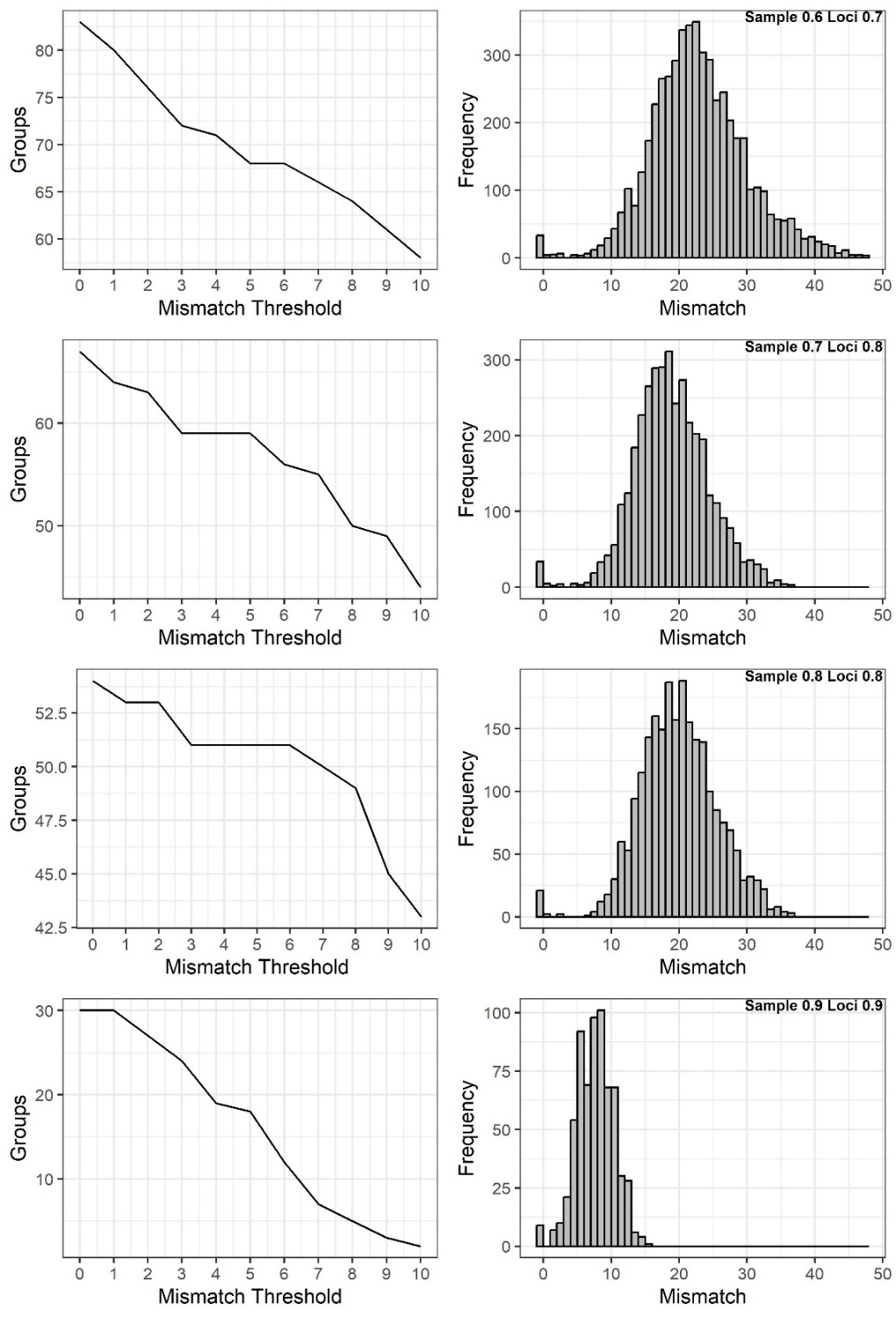


Figure S3: Number of groups of unique individuals at each mismatch threshold (‘*h*’; left column) and the frequency of each number of mismatches (right column), based on the SNP dataset, generated using ‘*ScatMatch*’. Each row represents a different combination of filtering parameters.


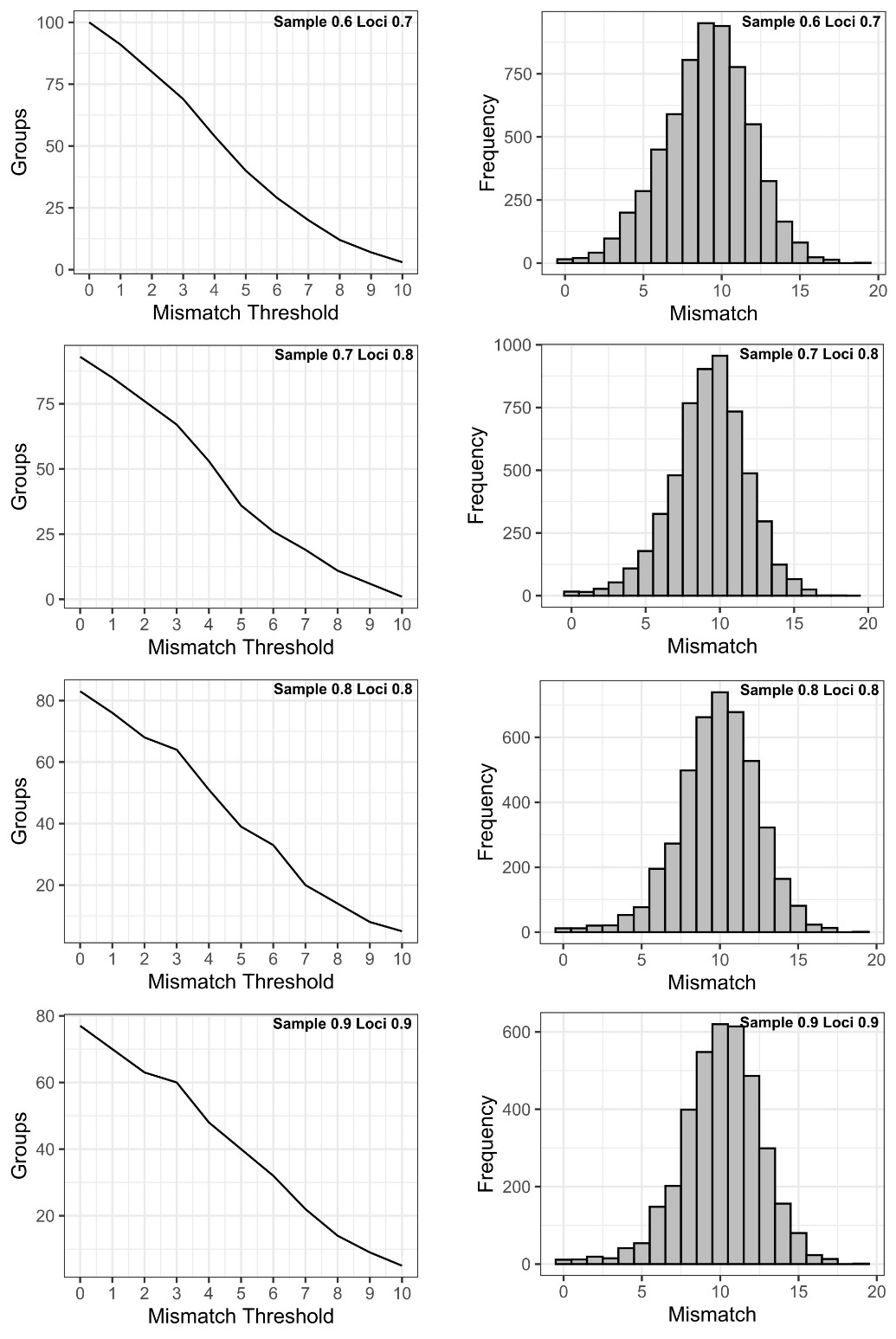


Figure S4: Number of groups of unique individuals at each mismatch threshold (‘*h*’; left column) and the frequency of each number of mismatches (right column), based on the microsatellite dataset, generated using ‘*ScatMatch*’. Each row represents a different combination of filtering parameters.


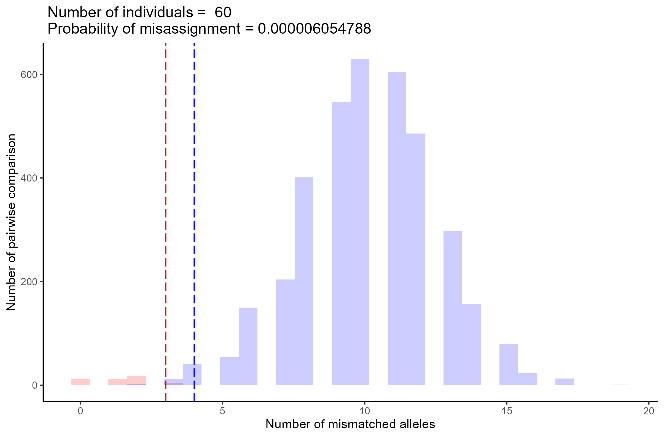

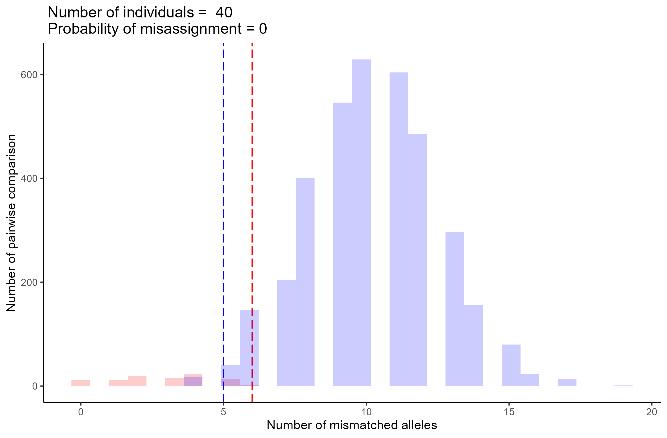


Figure S5: Misassignment graphs for different microsatellite mismatch thresholds generated in ‘*ScatMatch*’ (left *h*=3 and right *h*=5) showing the frequency of allelic mismatches where both samples have genotypes. The red dashed line represents the upper 0.995 percentile of pairwise mismatches ‘within’ groups and the blue dashed line represents the lower 0.005 percentile of mismatches ‘between’ groups.

Table S3: Assignment of scat DNA samples to unique individuals via ‘*ScatMatch*’, based SNP panel versus microsatellite genotyping.

| Sample | Matching | Split by MSATs | Split by SNPs | Grouped differently | Missing from one dataset |
| --- | --- | --- | --- | --- | --- |
| SC01311 | 1 |  |  |  |  |
| SC01370 | 1 |  |  |  |  |
| SC01067 | 1 |  |  |  |  |
| SC01079 |  |  |  |  | 1 |
| SC01080 | 1 |  |  |  |  |
| SC01141 | 1 |  |  |  |  |
| SC01313 |  | 1 |  |  |  |
| SC01314 |  |  | 1 |  |  |
| SC01326 |  |  | 1 |  |  |
| SC01316 | 1 |  |  |  |  |
| SC01095 |  |  | 1 |  |  |
| SC01325 | 1 |  |  |  |  |
| SC01330 |  |  | 1 |  |  |
| SC01351 | 1 |  |  |  |  |
| SC01382 | 1 |  |  |  |  |
| SC01333 |  |  | 1 |  |  |
| SC01334 | 1 |  |  |  |  |
| SC01336 |  |  | 1 |  |  |
| SC01065 |  |  | 1 |  |  |
| SC01342 |  |  |  |  | 1 |
| SC01346 |  |  |  | 1 |  |
| SC01349 | 1 |  |  |  |  |
| SC01350 |  |  |  |  | 1 |
| SC01371 |  |  |  | 1 |  |
| SC01353 | 1 |  |  |  |  |
| SC01360 | 1 |  |  |  |  |
| SC01357 | 1 |  |  |  |  |
| SC01366 |  |  | 1 |  |  |
| SC01396 | 1 |  |  |  |  |
| SC01358 |  |  | 1 |  |  |
| SC01373 |  |  | 1 |  |  |
| SC01359 |  |  | 1 |  |  |
| SC01361 |  |  |  | 1 |  |
| SC01377 |  |  |  |  | 1 |
| SC01378 | 1 |  |  |  |  |
| SC01074 | 1 |  |  |  |  |
| SC01098 | 1 |  |  |  |  |
| SC01112 | 1 |  |  |  |  |
| SC01384 | 1 |  |  |  |  |
| SC01387 | 1 |  |  |  |  |
| SC01389 |  |  |  |  | 1 |
| SC01392 |  | 1 |  |  |  |
| SC01402 | 1 |  |  |  |  |
| SC01063 | 1 |  |  |  |  |
| SC01064 | 1 |  |  |  |  |
| SC01071 |  | 1 |  |  |  |
| SC01073 |  | 1 |  |  |  |
| SC01077 |  | 1 |  |  |  |
| SC01082 | 1 |  |  |  |  |
| SC01084 | 1 |  |  |  |  |
| SC01091 |  | 1 |  |  |  |
| SC01094 | 1 |  |  |  |  |
| SC01097 |  |  |  |  | 1 |
| SC01099 | 1 |  |  |  |  |
| SC01100 |  |  | 1 |  |  |
| SC01101 |  |  | 1 |  |  |
| SC01102 |  |  |  |  | 1 |
| SC01108 | 1 |  |  |  |  |
| SC01156 | 1 |  |  |  |  |
| SC01105 | 1 |  |  |  |  |
| SC01106 |  |  |  |  | 1 |
| SC01135 |  |  |  | 1 |  |
| SC01109 | 1 |  |  |  |  |
| SC01111 |  | 1 |  |  |  |
| SC01117 | 1 |  |  |  |  |
| SC01121 |  |  |  |  | 1 |
| SC01122 |  |  |  |  | 1 |
| SC01123 | 1 |  |  |  |  |
| SC01126 | 1 |  |  |  |  |
| SC01131 | 1 |  |  |  |  |
| SC01164 | 1 |  |  |  |  |
| SC01130 | 1 |  |  |  |  |
| SC01132 |  | 1 |  |  |  |
| SC01133 |  | 1 |  |  |  |
| SC01137 |  |  |  |  | 1 |
| SC01140 |  | 1 |  |  |  |
| SC01142 | 1 |  |  |  |  |
| SC01146 |  | 1 |  |  |  |
| SC01150 | 1 |  |  |  |  |
| SC01159 | 1 |  |  |  |  |
| SC01160 | 1 |  |  |  |  |
| SC01161 | 1 |  |  |  |  |
| SC01162 | 1 |  |  |  |  |
| SC01168 |  |  | 1 |  |  |
| SC01169 | 1 |  |  |  |  |
| SC01170 |  |  |  |  | 1 |
| SC01171 |  | 1 |  |  |  |
| SC01329 |  |  |  |  | 1 |
| SC01343 |  |  |  |  | 1 |
| SC01139 |  |  |  |  | 1 |
| SC01369 |  |  |  |  | 1 |
| SC01394 |  |  |  |  | 1 |
| SC01070 |  |  |  |  | 1 |
| SC01107 |  |  |  |  | 1 |
| SC01088 |  |  |  |  | 1 |
| SC01089 |  |  |  |  | 1 |
| SC01090 |  |  |  |  | 1 |
| SC01103 |  |  |  |  | 1 |
| SC01166 |  |  |  |  | 1 |
|  | 45 | 12 | 14 | 4 | 24 |

Table S4: AIC comparison of null models fitted for closed population, multisession SECR model for mala density, using individual identification data obtained from SNP dataset and MSAT dataset. Parameters in these models relate to density (*D*), capture probability (g_0_), sigma (σ), and shape (z).

| Marker type | Rank | Model | Detection function | Parameters | dAICc |
| --- | --- | --- | --- | --- | --- |
| SNPs | 1 | D~1, g0~1, sigma~1, z~1 | Hazard rate | 4 | 0 |
|  | 2 | D~1, g0~1, sigma~1 | Exponential | 3 | 13.91 |
|  | 3 | D~1, g0~1, sigma~1 | Half normal | 3 | 26.47 |
| Microsatellites | 1 | D~1, g0~1, sigma~1, z~1 | Hazard rate | 4 | 0 |
|  | 2 | D~1, g0~1, sigma~1 | Exponential | 3 | 20.69 |
|  | 3 | D~1, g0~1, sigma~1 | Half normal | 3 | 38.51 |

Table S5: Mala density (*D*) and abundance for each session (year) and habitat type, based on the SNP dataset.

| Year | Habitat type | Density (*D*) | Standard error | Habitat area (ha) | Abundance |
| --- | --- | --- | --- | --- | --- |
| 2020 | Bare understory | 0.000 | 0.000 | 177.1 | 0.00 |
|  | Dense mulga over tuft grass | 0.048 | 0.031 | 215.6 | 10.43 |
|  | Dense shrubland over spinifex | 0.099 | 0.048 | 225.5 | 22.40 |
|  | Scattered shrubland over spinifex | 0.184 | 0.057 | 481.8 | 88.71 |
|  |  |  |  |  |  |
| 2021 | Bare understory | 0.000 | 0.000 | 177.1 | 0.00 |
|  | Dense mulga over tuft grass | 0.032 | 0.021 | 215.6 | 6.95 |
|  | Dense shrubland over spinifex | 0.066 | 0.033 | 225.5 | 14.93 |
|  | Scattered shrubland over spinifex | 0.123 | 0.041 | 481.8 | 59.14 |

Table S6: Mala density (*D*) and abundance for each session (year) and habitat type, based on the MSAT dataset.

| Year | Habitat type | Density (*D*) | Standard error | Habitat area (ha) | Abundance |
| --- | --- | --- | --- | --- | --- |
| 2020 | Bare understory | 0.000 | 0.000 | 177.1 | 0.000 |
|  | Dense mulga over tuft grass | 0.032 | 0.025 | 215.6 | 6.965 |
|  | Dense shrubland over spinifex | 0.109 | 0.046 | 225.5 | 24.525 |
|  | Scattered shrubland over spinifex | 0.159 | 0.045 | 481.8 | 76.413 |
|  |  |  |  |  |  |
| 2021 | Bare understory | 0.000 | 0.000 | 177.1 | 0.000 |
|  | Dense mulga over tuft grass | 0.018 | 0.014 | 215.6 | 3.814 |
|  | Dense shrubland over spinifex | 0.060 | 0.027 | 225.5 | 13.430 |
|  | Scattered shrubland over spinifex | 0.087 | 0.028 | 481.8 | 41.842 |

**References**

Bolton, B. L. and P. K. Latz (1978). "The Western Hare-Wallaby *Lagorchestes hirsutus* (Gould) (Macropodidae), in the Tanami Desert." Wildlife Research **5**(3): 285–293.

Clayton, J. (2012). Ecological and cultural aspects of the reintroduction of mala *Lagorchestes hirsutus* to Uluru PhD Thesis, University of New England.

Friend JA, Beecham BR (2004). Return to Dryandra: Western Shield review-February 2003. Conservation Science Western Australia. **5**(2):174.

Gibson, D.F., Johnson, K.A., Langford, D.G., Cole, J.R., Clarke, D.E., and Willowra Community. (1994). The rufous hare wallaby (*Lagorchestes hirsutus*): a history of experimental reintroduction in the Tanami Desert, Northern Territory.  Pp. 171-176. In: Reintroduction Biology of Australian and New Zealand Fauna. (Ed. S. Melody). Surrey Beatty & Sons, Chipping Norton, New South Wales.

Hardman, B. and D. Moro (2006). "Importance of diurnal refugia to a hare-wallaby reintroduction in Western Australia." Wildlife Research **33**(5): 355–359.

Johnson KA, Gibson DF, Langford DR & Cole JR (1996) Recovery of the Mala *Lagorchestes hirsutus*: a 30 year unfinished journey, in Stephens and Maxwell (eds), Back from the brink: refining the threatened species recovery process. Surrey Beatty & Sons.

Langford, D. C. and A. A. Burbidge (2001). "Translocation of the mala (*Lagorchestes hirsutus*) from the Tanami Desert, Northern Territory, to Trimouille Island, Western Australia." Australian Mammalogy **23**: 37–46.

Morris, K., C. Sims, K. Himbeck, et al. (2004). “Project Eden ‐ Fauna Recovery on Peron Peninsula, Shark Bay: Western Shield Review ‐ February 2003.” Conservation Science Western Australia **5**(2): 202–234.

Nally, S., Murphy, J., Barnwell, L., Kemp, L., Mala Recovery Team (in draft) Recovery Plan for the Mala (*Lagorchestes hirsutus* subsp.) and the Shark Bay rufous hare-wallaby (*Lagorchestes hirsutus* *bernieri*). Department of Climate Change, Energy, the Environment and Water, Canberra, Australian Capital Territory.

Richards, J. D. (2012). Rufous hare-wallaby (*Lagorchestes hirsutus*) national recovery plan.
